# Apoptosis, Pyroptosis, and Ferroptosis Conspiringly Induce Immunosuppressive Hepatocellular Carcinoma Microenvironment and γδ T-Cell Imbalance

**DOI:** 10.3389/fimmu.2022.845974

**Published:** 2022-04-04

**Authors:** Yi Hu, Dan Chen, Minjing Hong, Jing Liu, Yijia Li, Jianlei Hao, Ligong Lu, Zhinan Yin, Yangzhe Wu

**Affiliations:** ^1^Guangdong Provincial Key Laboratory of Tumour Interventional Diagnosis and Treatment, Zhuhai Institute of Translational Medicine, Zhuhai People’s Hospital Affiliated with Jinan University, Jinan University, Zhuhai, China; ^2^Microbiology and Immunology Department, School of Medicine, Faculty of Medical Science, Jinan University, Guangzhou, China; ^3^The Biomedical Translational Research Institute, Faculty of Medical Science, Jinan University, Guangzhou, China

**Keywords:** apoptosis, pyroptosis, ferroptosis, immunosuppressive HCC TME, γδ T-cell imbalance

## Abstract

Hepatocellular carcinoma (HCC) is highly malignant and prone to metastasize due to the heterogeneous and immunosuppressive tumor microenvironment (TME). Programmed cell deaths (PCDs) including apoptosis, ferroptosis, and pyroptosis routinely occur in the HCC TME and participate in tumorigenesis. However, how apoptosis, ferroptosis, and pyroptosis are involved in constructions of the immunosuppressive TME and their underlying cross-talk remains to be further unveiled. In this work, we deciphered the immunosuppressive landscape of HCC TME, which demonstrated high expressions of inhibitory checkpoint molecules and infiltration of protumor immune cells but low infiltration of antitumor effector immune cells. Further investigations unequivocally revealed that marker genes of apoptosis, ferroptosis, and pyroptosis are closely correlated with expressions and infiltrations of inhibitory checkpoint molecules and immune cells and that higher “-optosis” links to poorer patient prognosis. Notably, such three types of “-optosis” interact with each other at both the gene and protein levels, suggesting that they conspiringly induce the establishment of the immunosuppressive HCC TME. Interestingly, examinations of circulating γδ T cells in HCC patients revealed a noticeable dysfunction phenotype. The strikingly elevated ratio of the Vδ1^+^ versus the Vδ2^+^ subset suggested that the Vδ1^+^/Vδ2^+^ ratio would be a potential biomarker for the diagnosis and prognosis in HCC patients. Altogether, this work thoroughly decrypted the underlying correlations between apoptosis, ferroptosis, and pyroptosis and the formation of immunosuppressive HCC TME and, meanwhile, indicated that allogeneic Vδ2^+^ γδ T-cell transfer would be a promising adjuvant strategy for renormalizing circulating γδ T cell and thus achieving sound clinical efficacy against HCC.

## Introduction

The liver is a major metabolic organ and plays a crucial role in all metabolism processes in the body. In certain circumstances, such as alcohol abuse, virus infection, and drugs, liver inflammation will occur and can deteriorate into hepatic fibrosis, hepatic cirrhosis, and eventually liver cancer. Generally, tumorigenesis is a result induced by complicated events, and one determinant factor is the long-term suppressive or dysfunctional immunity of hosts. Hepatocellular carcinoma (HCC) is well-known for its high relapse rate and poor long-term prognosis. Current routine treatments of HCC rely heavily on chemotherapeutic approaches (e.g., sorafenib) but have unsatisfactory results. Fortunately, immunotherapy strategies (1) including immune checkpoint blockade (ICB) (2) and immune cell therapy (3–5) provide new opportunities for HCC patients. For example, PD1 blockade therapy is now covered for free by the National Healthcare Security System of China and benefits the increasing HCC population. Nevertheless, further decoding of the HCC tumor microenvironment (TME), especially the immune landscape, will benefit the development of immunotherapies. For HCC, how the immunosuppressive microenvironment is triggered and eventually established remains to be further illustrated.

The excessive proliferation nature of cancer cells and the antitumor immunity of the host unavoidably lead to numerous types of cell death, mainly including programmed (e.g., apoptosis) and non-regulated (e.g., necrosis) cell deaths. Currently, intensively investigated programmed cell deaths (PCDs) in HCC TME include apoptosis, ferroptosis, and pyroptosis. Apoptosis belongs to the non-immunogenic cell death and shall not induce detectable immune responses under normal circumstances. On the other hand, immunogenic deaths, ferroptosis, and pyroptosis can result in immune responses due to the release of various intracellular contents, most acting as pro-inflammatory signals. Though the role of ferroptosis and pyroptosis in HCC prognosis had been previously discussed (6, 7), how non-immunogenic apoptosis as well as immunogenic ferroptosis and pyroptosis cross-talk at the gene and protein levels and collaboratively contribute to the development of the immunosuppressive HCC TME remain to be further addressed.

In the HCC TME, there are various types of infiltrated immune cells, including lymphoid and myeloid linage cells. Among them, γδ T cell occupies 6.8%~34% CD3^+^ T cell in the liver (8, 9) and plays crucial roles in liver protection from virus infection (10) and tumorigenesis (11, 12). Intra-tumoral γδ T cell was proposed to be one of the best positive prognosis markers for pan-cancers (13). However, evidence indicated that the two major subsets of γδ T cell, namely, Vδ1^+^ and Vδ2^+^ subsets, possess contradictory roles in antitumor immunity. The Vδ1^+^ subset tends to perform a Treg-like function in the context of TME, which expresses high levels of inhibitory surface markers like CD73 (14) and CD39 (15), as well as cytokines such as IL10 (15) and TGFβ (16). The Vδ2^+^ subset, however, is the early source of IFNγ (17, 18) and performs cytotoxic functions against transformed cells (19). For years, we have mainly focused on the Vδ2^+^ subset; most importantly, we innovatively proved the safety and efficacy of the allogeneic Vδ2^+^ γδ T cells in a series of clinical studies (4, 5, 20). Therefore, the primary scientific question we want to illustrate is the performance of the Vδ2^+^ γδ T cell in the context of HCC TME. Here we not only applied bioinformatics to evaluate the correlations between γδ T-cell infiltration and three types of cell death (apoptosis, ferroptosis, and pyroptosis) but also statistically evaluated the imbalance of circulating γδ T-cell subsets in HCC patients.

Together, our present work revealed that the HCC TME is globally immunosuppressive, and three types of “-optosis” (apoptosis, ferroptosis, and pyroptosis) play crucial roles in creating the immunosuppressive TME and lead to the adverse prognosis of HCC patients. Moreover, we found that the proportion of the Vδ1/Vδ2 subsets of circulating γδ T cell is functionally imbalanced in HCC patients, suggesting that the adoptive transfer of allogeneic Vδ2^+^ γδ T cells from healthy donors might benefit patients. We further hypothesize that by taking advantage of apoptosis, ferroptosis, and pyroptosis, immune cell-based adjuvant therapy (e.g., Vδ2^+^ γδT) might lead to a better prognosis than chemotherapy alone or chemotherapy combined with checkpoint blockade.

## Methods and Materials

### γδ T-Cell Subset Proportioning in the Healthy Population and Hepatocellular Carcinoma Patients

To reveal the proportional difference in the two subsets of γδ T cells from peripheral blood mononuclear cells (PBMCs) between the healthy population and HCC patients, the raw immune-phenotyping data of these two populations provided by the Shuangzhi Purui Medical Laboratory Co., Ltd. (Wuhan, China) were obtained and analyzed. The healthy population consisted of 170 disease-free adults (age range, 26–72; gender includes both female and male). The HCC population consisted of 42 patients (age range, 30–71; diagnosis, HCC I to IV including both primary and recurrence; gender includes both female and male). The antibodies used included anti-human CD3, anti-human TCR Vδ1, and anti-human TCR Vδ2 antibodies (BioLegend, San Diego, CA, USA). All examinations were conducted using BD FACSCanto cytometry (BD Biosciences, San Jose, CA, USA).

### Online Databases and Bioinformatics Analysis

Tumoral mRNA-sequencing data (level 3) and corresponding clinical information of 371 HCC patients were obtained from The Cancer Genome Atlas (TCGA) dataset (https://portal.gdc.cancer.gov/) by following the related guidelines and policies. The mRNA-seq data of 50 paired peri-tumor tissue samples, together with 226 healthy liver tissue samples from the GTEx database (GTEx V8, https://gtexportal.org/home/datasets), were used as the control group, which described donors’ clinical information including gender, race, age, clinical stages, therapy history, and biospecimen collection. Protein–protein interaction was analyzed using the online database (https://string-db.org/). The R Project for Statistical Computing version 4.0.3 was used for graph plotting and analysis unless specified otherwise. In our analysis, HCC patients were divided into three groups (I, II, and III–IV) according to the clinical stage (pTNM) due to the limited amount of stage IV patients. To analyze the multi-gene correlation, the mRNA-seq dataset from NIH National Cancer Institute (https://tcga-data.nci.nih.gov/tcga/) was also included. The R software packages ggstatsplot and pheatmap were respectively used to produce two-gene and multi-gene correlation maps. Spearman’s analysis was used to describe the correlation.

As for ferroptosis analysis, the related marker genes were derived from previously published data (21). The marker genes for apoptosis were adopted from http://biocc.hrbmu.edu.cn/CancerSEA/home.jsp. The marker genes for pyroptosis were from published work (22). Then R packages ggplot2 and pheatmap were used for data visualization. Based on marker genes of apoptosis, ferroptosis, and pyroptosis, we wanted to know whether samples could be clustered into subgroups. The R packages ConsensusClusterPlus and pheatmap were used to generate clustering heatmaps. Moreover, the Kaplan–Meier survival analysis with log-rank test was used to plot survival curves among groups; p-values and hazard ratio (HR) with 95% CI were generated by log-rank tests and univariate Cox proportional hazards regression. For correlation between single gene and survival, R packages ggrisk, survival, survminer, glmnet, and timeROC were applied for analysis.

To evaluate immune cell infiltration in the context of apoptosis, ferroptosis, and pyroptosis, the R package immunedeconv was applied, which integrates six classes of algorithms, including TIMER, xCell, MCP-counter, CIBERSORT, EPIC, and quanTIseq. Meanwhile, the gene expression of inhibitory checkpoint molecules PDCD1(PD1), CD274(PDL1), PDCD1LG2(PDL2), CTLA4, LAG3, TIGIT, HAVCR2(TIM3), and SIGLEC15 in HCC tissues were intensively inspected. To analyze the correlation between “-optosis” related marker genes and 15 checkpoint molecules, both stimulatory checkpoint molecules (CD27, CD28, CD40, DNAM1, ICOS, and TNFRSF9) and inhibitory checkpoint molecules (CD274, CTLA4, HAVCR2, LAG3, PDCD1, PDCD1LG2, TIGIT, SIGLEC15, and BTLA) were included. To assay ICB among HCC subgroup samples, the potential ICB response was predicted with the Tumor Immune Dysfunction and Exclusion (TIDE) algorithm (23). p-Value <0.05 was considered statistically significant.

## Results

### Hepatocellular Carcinoma Tumor Microenvironment Is Highly Immune Suppressed

**Figure 1:**
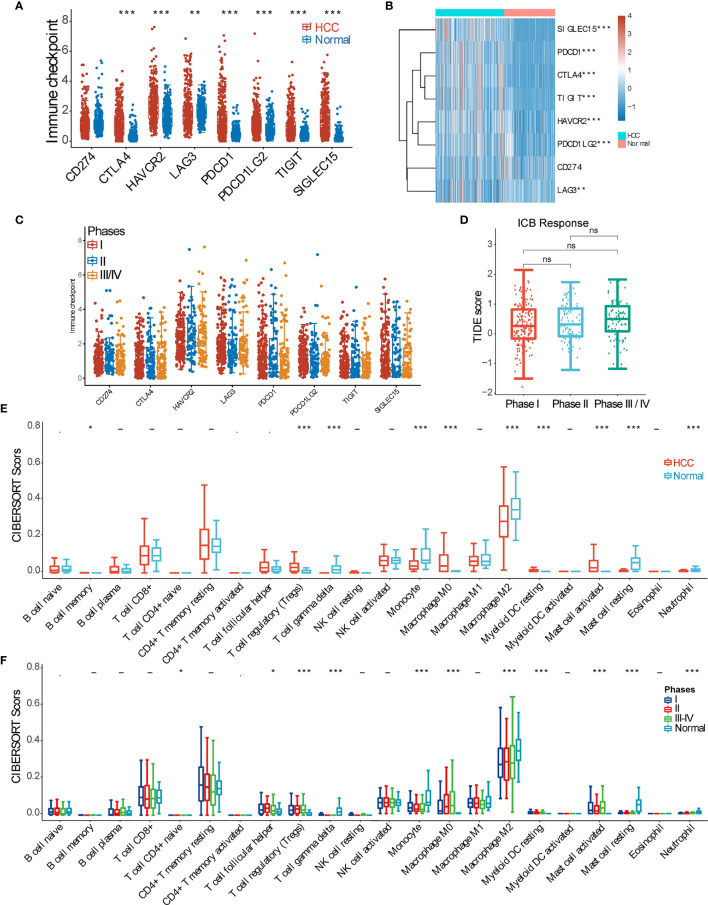
Overview of the immune microenvironment of hepatocellular carcinoma (HCC) tissue. **(A)** Expression difference of inhibitory checkpoint molecules (CD274(PDL1), CTLA4, HAVCR2(TIM3), LAG3, PDCD1(PD1), PDCD1LG2(PDL2), TIGIT, and SIGLEC15) between HCC and normal tissue samples. **(B)** Heatmap of inhibitory checkpoint molecule expression. **(C)** Expression of inhibitory checkpoint molecules in HCC samples of pTNM stages I, II, and III–IV. **(D)** Tumor Immune Dysfunction and Exclusion (TIDE) score analysis was used to predict the responses to immune checkpoint blockade (ICB) of HCC patients at pTNM stages I, II, and III–IV. **(E)** CIBERSORT score of various immune cell infiltrations in HCC and normal tissue samples. **(F)** CIBERSORT score of various immune cell infiltrations among HCC samples of pTNM stages I, II, and III–IV. *p < 0.05; **p < 0.01; ***p < 0.001; ‘-’, ns, no statistical significance.

To comprehensively inspect the immune microenvironment of HCC TME, we firstly compared gene expression of immune inhibitory checkpoint molecules between tumor tissues and normal (peri-tumor) tissues, including CD274, CTLA4, HAVCR2, LAG3, PDCD1, PDCD1LG2, TIGIT, and SIGLEC15. It showed that all inhibitory checkpoint molecules except for CD274 are significantly upregulated in the HCC tumor group (**Figures 1A, B**). Then, 371 HCC samples were divided into 3 groups based on clinical stage (pTNM), and the checkpoint molecules were further compared among the 3 subgroups. It turned out that all checkpoint molecules are not statistically different among subgroups (**Figure 1C**). Further analysis revealed that HCC patients with different clinical stages respond to ICB therapy similarly (**Figure 1D**). We further checked the difference of immune cell infiltration between the HCC and normal groups by CIBERSORT score comparisons. We found that in the HCC group, scores of regulatory T cells (Tregs), resting macrophages M0, resting myeloid dendritic cells (DCs), and activated mast cells are strikingly increased. On the contrary, CIBERSORT scores of infiltrated γδ T cells, monocytes, macrophages M2, and resting mast cells are all depressed (**Figure 1E**). Furthermore, we inspected tissue infiltration of these immune cells among subgroups according to clinical stages (I, II, III–IV, and normal) and observed a similar pattern (**Figure 1F**) to **Figure 1E**.

### Apoptosis Contributes to Establishing the Immunosuppressive Hepatocellular Carcinoma Tumor Microenvironment

**Figure 2:**
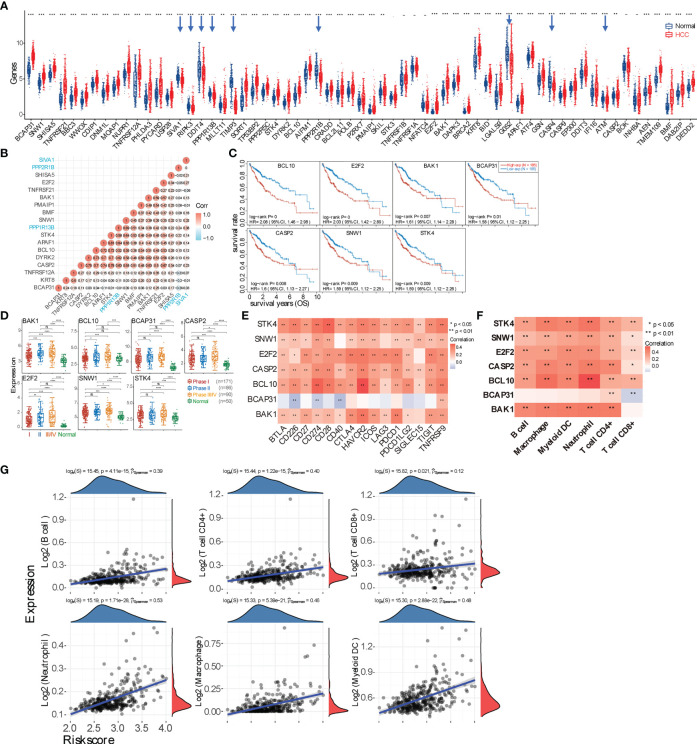
Evaluating the underlying correlation between apoptosis and immune microenvironment. **(A)** Expression spectrum of apoptosis marker genes in hepatocellular carcinoma (HCC) tumor tissue. Downregulated genes are highlighted by blue arrows. **(B)** Multi-gene correlation map generated using the genes with significant (p < 0.05) correlation with the overall survival (OS) rate. A positive value represents a positive correlation; otherwise, a negative correlation. The larger correlation value means the better correlation between two genes. The three genes labeled with blue color are downregulated genes in panel **(A, C)** Survival curves of 7 genes (BCL10, E2F2, BAK1, BCAP31, CASP2, SNW1, and STK4) that negatively correlate with prognosis (p < 0.01). **(D)** Expression difference of those 7 genes in HCC tumor tissues among patients with stage I, II, or III–IV. **(E)** Correlation analysis between 7 marker genes and checkpoint molecules. Only BCAP31 negatively correlates with checkpoint molecules. **(F)** Correlation between 7 marker genes and major types of immune cell infiltration. **(G)** Spearman’s correlation analysis between the risk score of the prognostic model (calculated from the 7 marker genes) and immune score (based on TIMER). The horizontal axis represents the risk score of the prognostic model; the vertical axis represents the immune score. The density curve (red) on the right represents the distribution trend of the immune score, and the dark-blue density curve on the top represents the distribution trend of the risk score. The p-value and correlation coefficient $\left(\hat{\text{p}}\text{Spearman}\right)$ are shown at the top of the respective graph. *p < 0.05; **p < 0.01; ***p < 0.001; ****p < 0.0001; ‘-’, ns, no statistical significance.

Due to the excessive proliferation of HCC tumor cells and corresponding antitumor immunity and because cancer cell apoptosis routinely occurs in the TME, we first checked the expression differences of 63 apoptosis marker genes between HCC and normal tissues. We can see that 48 apoptosis-related marker genes are significantly upregulated in the HCC group (9 downregulated genes highlighted by blue arrows and 6 genes with no statistical difference) (**Figure 2A**). We then analyzed the correlation between each of the 63 marker genes and the overall survival (OS) rate and found that only 22 apoptotic genes are significantly correlated with the OS (p < 0.05). Specifically, 21 genes have negative correlations, while only one gene (PPP2R1B) has a positive correlation (**Supplementary Figure 2A**). Afterward, the multi-gene correlation among these 22 genes was calculated, as shown in **Figure 2B**. Moreover, we intensively analyzed those genes that have a better correlation with OS (p ≤ 0.01), including BCL10, E2F2, BAK1, BCAP31, CASP2, SNW1, and STK4. It shows that higher expressions of these 7 genes lead to poorer prognoses (**Figure 2C**). Further analysis revealed that expressions of these 7 genes among different clinical stages (I, II, and III–IV) are consistently and greatly upregulated as compared with the normal group, and the expression level dramatically increases from stage I to stage III–IV (**Figure 2D**). We then found that six of these seven genes (except BCAP31) are positively correlated with the expression level of checkpoint molecules and immune cell infiltration (**Figures 2E, F**; **Supplementary Figure 2B**).

Based on the expressions of those 22 genes that are correlated with OS, 371 HCC samples were further divided into 3 subgroups using the R package ConsensusClusterPlus (**Supplementary Figure 2C**), and the clinical information of these 3 groups is shown in the table. It shows that the expressions of most checkpoint molecules (except SIGLEC15) are significantly elevated in “G2” and “G3” compared with “G1.” CIBERSORT scores indicated that infiltrations of naïve B cells, CD8^+^ T cells, naïve CD4^+^ T cells, resting NK cells, monocytes, macrophage M1, and activated mast cells are statistically different among the three groups (p < 0.01). Further TIDE score analysis revealed that “G2” and “G3” have better ICB responses. However, OS probability shows no statistical difference among the three subgroups. Furthermore, 7 genes with a p ≤ 0.01 correlation with OS were used to construct a prognostic signature model using the least absolute shrinkage and selection operator (LASSO) Cox regression model. The obtained Riskscore equals (0.072) * *BAK1* + (0.1257) * *BCAP31* + (0.0895) * *BCL10* + (0.2596) * *E2F2* + (0.1424) * *SNW1* (**Supplementary Figure 2D**). Based on this prognostic model, the correlation between the Riskscore and immune cell infiltration was further visualized (**Figure 2G**).

### Ferroptosis Is Involved in Constructing the Immunosuppressive Hepatocellular Carcinoma Tumor Microenvironment

**Figure 3:**
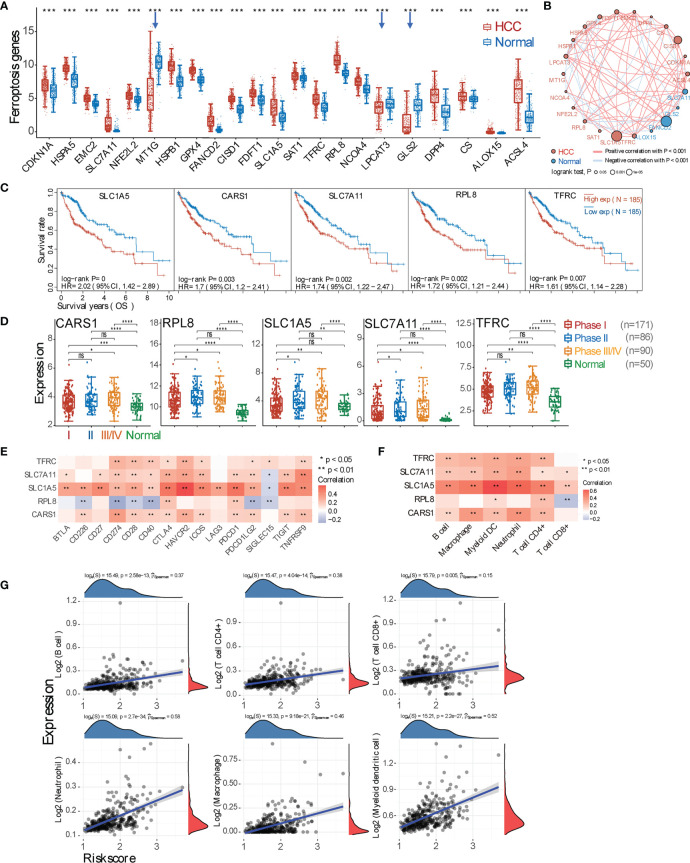
Marker gene expression of ferroptosis and its correlation with tumor microenvironment (TME) immune profile. **(A)** Expression comparison of ferroptosis marker genes between hepatocellular carcinoma (HCC) and normal tissues. The downregulated genes in HCC tissues are highlighted by blue arrows. **(B)** Gene relationship. Each circle represents a gene: the red one means positive, while the blue one represents negative correlation. The different sizes of the circle mean the different log-rank p-value. **(C)** Representative overall survival curves of 5 genes (SLC1A5, CARS1, SLC7A11, RPL8, and TFRC) that are negatively and significantly correlated with prognosis (p < 0.01). **(D)** Expression of these 5 marker genes in tumor tissues from pTNM stage I, II, or III–IV patients. **(E)** Correlation analysis between 5 marker genes and checkpoint molecules. **(F)** Correlation between 5 marker genes and major types of immune cell infiltration. **(G)** Spearman’s correlation analysis between the risk score of the prognostic model (calculated from the 5 marker genes) and the immune score. The horizontal axis represents the risk score of the prognostic model; the vertical axis represents the immune score. The density curve (red) on the right represents the distribution trend of the immune score, and the dark-blue density curve on the top represents the distribution trend of the risk score. The p-value and correlation coefficient $\left(\hat{\text{p}}\text{Spearman}\right)$ are shown at the top of the respective graph. *p < 0.05; **p < 0.01; ***p < 0.001; ****p < 0.0001; ‘-’, ns, no statistical significance.

Given that ferroptosis can be conditionally triggered by chemotherapy or can spontaneously occur under oxidative stress in the HCC microenvironment, we then checked the overall expressions of ferroptosis marker genes between HCC and normal samples. It shows 19 upregulated and 3 downregulated genes in HCC (**Figure 3A**), and the gene correlation in HCC and normal tissues is shown in **Figure 3B** and **Supplementary Figure 3A**. We then calculated how expressions of these genes would affect patient prognosis, and we found that higher expressions of genes SLC1A5, CARS1, SLC7A11, RPL8, and TFRC lead to poorer OS (p < 0.01) (**Figure 3C** and **Supplementary Figure 3B**). Moreover, the expressions of these 5 genes are gradually enhanced from tumor stage (pTNM) I to III/IV (**Figure 3D**). Subsequently, the correlation between these 5 genes and checkpoint molecules or immune cells was analyzed, as shown in **Figures 3E, F**. Moreover, HCC samples were also sub-clustered into 4 groups based on the expression of 22 ferroptosis marker genes, showing the difference in immune cell infiltration as well as expressions of inhibitory checkpoint molecules among subgroups (**Supplementary Figure 3C**). TIDE score revealed that these 4 subgroups respond to ICB differently, and the OS probability among groups is statistically different (p < 0.001). Furthermore, a prognostic signature model was constructed, and the obtained Riskscore equals (0.1218) * *CARS1* + (0.0359) * *RPL8* + (0.0095) * *TFRC* + (0.1585) * *SLC7A11* + (0.1652) * *SLC1A5* (**Supplementary Figure 3D**), which was used to calculate the correlation between the Riskscore and immune cell infiltration (**Figure 3G**).

### Pyroptosis Conspires in Creating the Immunosuppressive Hepatocellular Carcinoma Tumor Microenvironment

**Figure 4:**
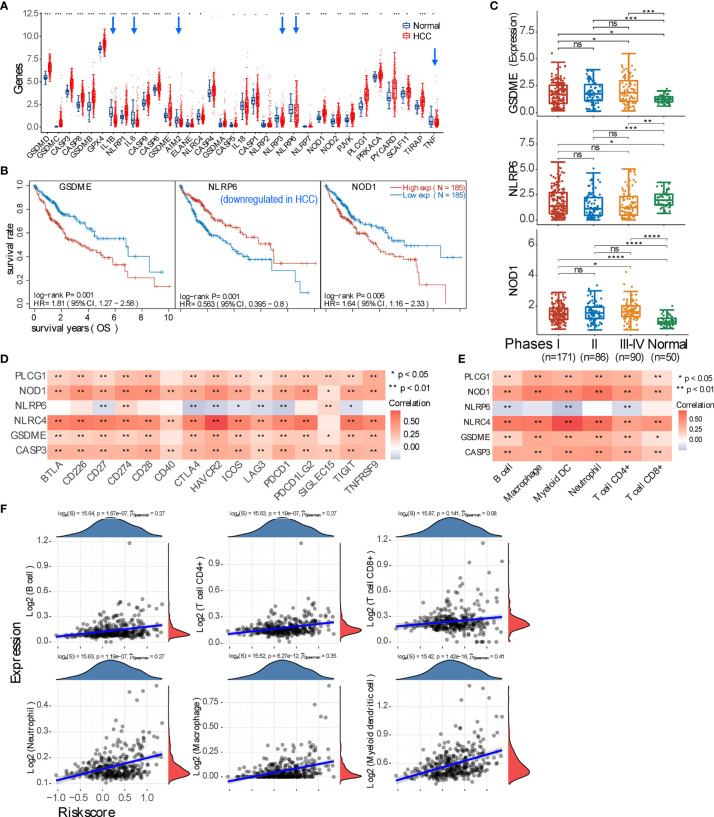
Expression of pyroptosis marker genes and their immune correlation in the context of hepatocellular carcinoma (HCC). **(A)** Expression profile of pyroptosis marker gene in HCC and normal tissue. The downregulated genes in HCC are pointed by blue arrows. **(B)** Three marker genes are significantly correlated with prognosis (p < 0.01); specifically, NLRP6 positively correlates with the overall survival of HCC patients. **(C)** Expression comparison of these 3 genes (GSDME, NLRP6, and NOD1) in HCC patients with pTNM stage I, II, or III–IV. **(D, E)** Correlation analysis between all genes that have p < 0.05 correlation with prognosis and checkpoint molecules, or immune cell infiltration. **(F)** Spearman’s correlation analysis between the risk score of the prognostic model (calculated from the 3 marker genes) and immune score. *p < 0.05; **p < 0.01; ***p < 0.001; ****p < 0.0001; ‘-’, ns, no statistical significance.

In addition to ferroptosis, pyroptosis is another type of inflammatory cell death. It is thus interesting to unveil the relationship between pyroptosis and the immune response in the HCC microenvironment. We found that, among 33 marker genes, 21 genes are upregulated, 6 genes are downregulated, and 6 genes have no statistical difference (**Figure 4A**). Further analysis revealed that only 6 genes (CASP3, GSDME, NLRC4, NLRP6, NOD1, and PLCG1) are statistically correlated with the OS rate of HCC patients (p < 0.05) (**Figure 4B** and **Supplementary Figure 4A**), and only GSDME, NLRP6, and NOD1 are significantly correlated with the OS (p < 0.01). Moreover, genes GSDME and NOD1 expressed higher in pTNM stage III–IV compared with stages I and II (**Figure 4C**). Further evaluation revealed that these 6 genes except NLRP6 are all positively correlated with checkpoint molecule expression and immune cell infiltration (**Figures 4D, E**). Additionally, all HCC samples can be grouped into two subclusters based on pyroptosis marker genes (**Supplementary Figure 4B**), and then expressions of checkpoint molecules, immune cell infiltration (TIMER and CIBERSORT scores), ICB, and the OS probability between two subgroups were all deciphered (**Supplementary Figures 4C, D**). The prognostic signature model was also generated by using the genes with p < 0.01, and the Riskscore equals (0.2413) * GSDME + (−0.2142) * NLRP6 + (0.0892) * NOD1, and the correlation between the Riskscore and immune cell infiltration was obtained (**Figure 4F** and **Supplementary Figure 4E**).

### Cross-Talk of Apoptosis, Ferroptosis, and Pyroptosis in Hepatocellular Carcinoma Tumor Microenvironment

**Figure 5:**
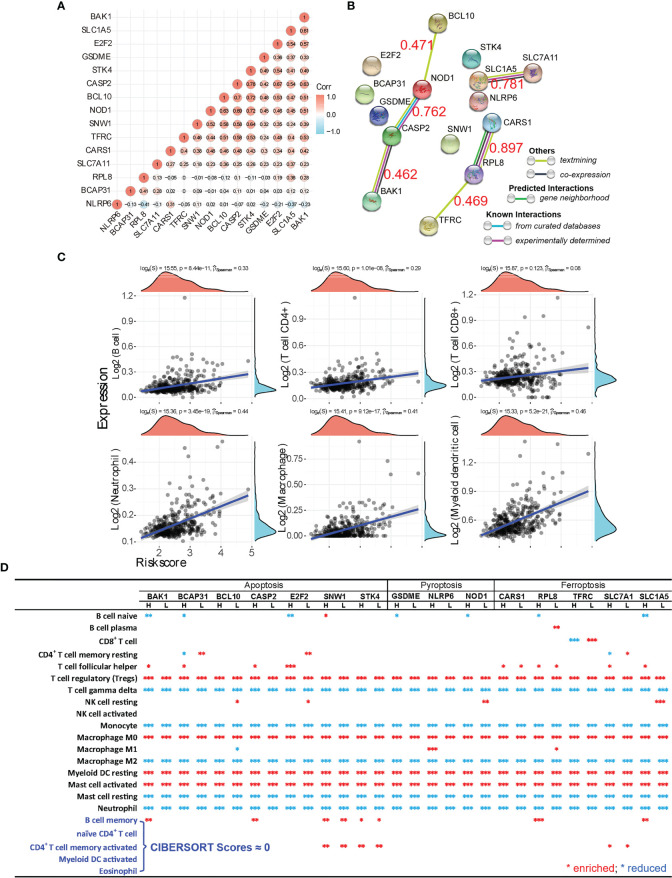
Marker gene cross-talking analysis of apoptosis, ferroptosis, and pyroptosis. **(A)** The gene-gene correlation of all “-optosis” marker genes that have significant correlations with the overall survival (OS) (p < 0.01) is analyzed. The correlation coefficient is shown on each circle, and the red circle refers to a positive correlation, while the blue one means a negative correlation. **(B)** Using the analysis tool provided by the STRING database, the interaction of protein corresponding to each “-optosis” marker gene was calculated as well, and the correlation coefficient between genes is marked as red numeric. **(C)** Spearman’s correlation analysis between the risk score of the prognostic model (calculated from the 15 “-optosis” marker genes, BCL10, E2F2, BAK1, BCAP31, CASP2, SNW1, STK4, SLC1A5, CARS1, SLC7A11, RPL8, TFRC, GSDME, NLRP6, and NOD1) and immune score. **(D)** Using the CIBERSORT analysis, the comprehensive relationship between the expression of each “-optosis” marker gene (p < 0.01 correlating with the OS) and immune cell infiltration is revealed. The red asterisk means enriched infiltration, and the blue one means reduced infiltration of immune cells. *p < 0.05; **p < 0.01; ***p < 0.001.

In the context of HCC TME, apoptosis, ferroptosis, and pyroptosis occur in an interconnecting manner, and their cross-talk may exist at both the gene and protein levels. We thus evaluated those marker genes that have a p < 0.01 correlation with the OS rate of HCC patients and found that these three types of “-optosis” genes do show cross-talk at the genomic level (**Figure 5A** and **Supplementary Figure 5A**). For example, NOD1 (pyroptosis gene) associates with both apoptosis genes (BCL10, CASP2, STK4, E2F2, and BAK1) and ferroptosis genes (TFRC, SLC1A5, and CARS1), and apoptosis gene BCL10 connects with pyroptosis genes (NOD1 and GSDME) and ferroptosis genes (SLC1A5, TFRC, CARS1, and SLC7A11). The protein–protein correlation analysis (STRING database, https://string-db.org/) further supports the analysis at the mRNA level, clearly showing the existing interactions among apoptosis, ferroptosis, and pyroptosis at the protein levels (**Figure 5B** and **Supplementary Figure 5B**). Next, we examined the protein–protein interactions between 15 checkpoint molecules and the “-optosis” genes that significantly correlate (p < 0.01 and <0.05, respectively) with the OS rate (**Supplementary Figures 5C, D**); the result indicated that only 5 checkpoint molecules (CD27, CD28, CD40, CTLA4, and PDL1) correlate with “-optosis” proteins. Afterward, 15 “-optosis” genes (significantly correlating with the OS rate, p < 0.01) were used to construct the prognostic signature model, and the obtained Riskscore (**Supplementary Figure 5E**) was correlated with immune cell infiltration (**Figure 5C**), indicating positive correlations with 6 main types of immune cells and specifically relative higher correlations with neutrophils, macrophages, and myeloid DCs (p_Spearman_ > 0.4).

### Apoptosis–Ferroptosis–Pyroptosis Collaboratively Induces Immunosuppressive Hepatocellular Carcinoma Tumor Microenvironment

**Figure 6:**
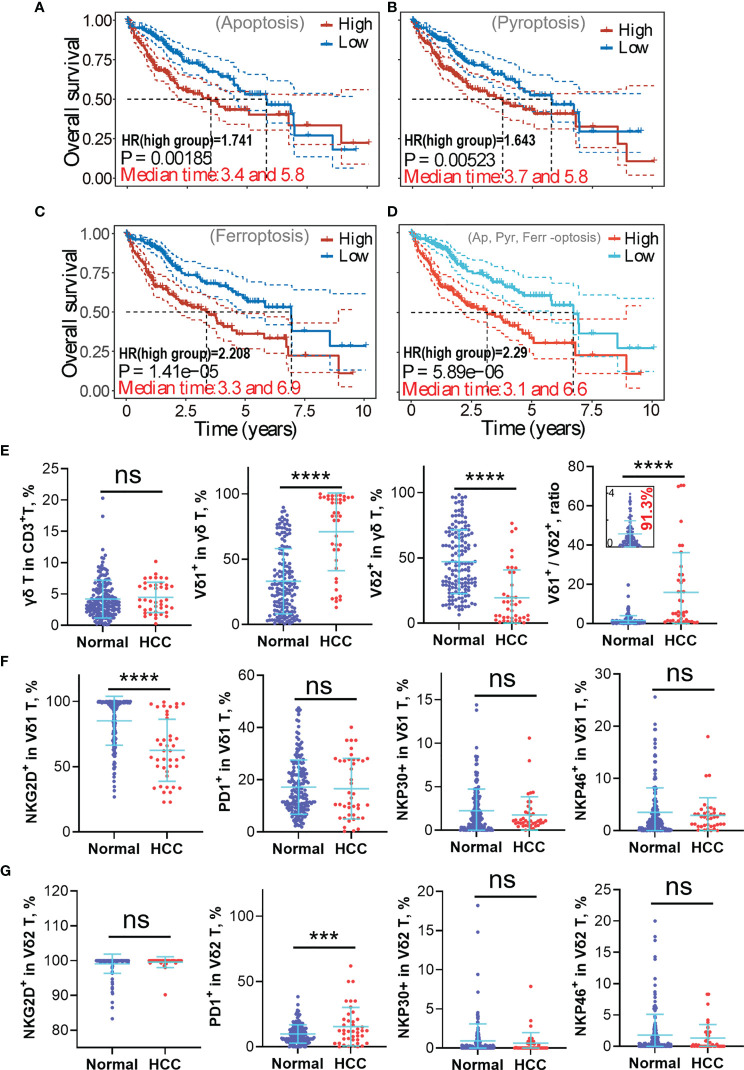
High expressions of apoptosis, ferroptosis, and pyroptosis-related marker genes in hepatocellular carcinoma (HCC) lead to poorer prognosis and implicate γδ T-cell depletion, particularly Vδ2^+^ γδ T-cell depletion. **(A–C)** The “-optosis” marker genes were used to create the prognostic signature model using the least absolute shrinkage and selection operator (LASSO) Cox regression model (cited from **Supplementary Figures 2D, 3D, 4E**), the correlations between the Riskscore and overall survival (OS) are graphically shown here. It shows higher expression of marker genes positively correlates with higher risk coefficient (HR > 1), leading to poorer clinical outcome. **(D)** Correlation between the Riskscore (generated using 15 “-optosis” marker genes) and the OS (cited from **Supplementary Figure 5E**). Higher Riskscore leads to a poorer prognosis. **(E)** Circulating γδ T-cell phenotypes from the healthy population and HCC patients were analyzed using flow cytometry, including the proportion of γδ T cell in CD3^+^ T cells, Vδ1^+^ subset in γδ T cell, Vδ2^+^ subset in γδ T cell, and the Vδ1^+^/Vδ2^+^ ratio. **(F)** Functional phenotypes of circulating Vδ1^+^ γδ T cell, including expressions of NKG2D, PD1, NKP30, and NKP46. **(G)** Functional phenotypes of circulating Vδ2^+^ γδ T cell, including expressions of NKG2D, PD1, NKP30, and NKP46. ns, no statistical significance; ***p < 0.001; ****p < 0.0001.

Since cross-talk of apoptosis, ferroptosis, and pyroptosis was demonstrated at both the gene and protein levels, it is interesting to decode whether they conspiringly participate in inducing the immune-suppressiveness of HCC TME. We thus analyzed how each marker gene (p < 0.01 correlation with the OS) correlated with immune cell infiltration (**Figure 5D**). The result unequivocally indicates that tumor-infiltrating Tregs, macrophage M0, resting myeloid DCs, and activated mast cells are all significantly enriched in the context of expressions of the listed marker genes. Meanwhile, γδ T cell, monocytes, macrophage M2, resting mast cells, and neutrophils are all significantly suppressed. Importantly, both enriched and reduced immune cells significantly (p < 0.001) correlate with the “-optosis” marker genes regardless of their high or low expression in HCC tissue compared with normal tissue. Additionally, OS analysis generated by the prognostic signature model shows that higher expressions of marker genes (p < 0.01 correlation with the OS) of apoptosis, ferroptosis, and pyroptosis lead to poorer prognosis both individually (**Figures 6A–C**) and collaboratively (**Figure 6D**) (**Supplementary Figures 2D, 3D, 4E,** and **5E**). These results again suggest that apoptosis, ferroptosis, and pyroptosis altogether participate in creating the immunosuppressive HCC TME.

### Immunosuppressive Hepatocellular Carcinoma Tumor Microenvironment Induces γδ T-Cell Imbalance

Since the liver is one of the richest sources of tissue-resident γδ T cells, and our previous works unequivocally proved that adoptive transfer therapy of allogeneic Vδ2^+^ γδ T cells could safely and efficiently help control liver cancer progression or even achieve complete remission (4, 5), we thus focused on exploring how γδ T cell is repressed in the HCC TME (**Figure 5D**). We tried to inspect the underlying difference of circulating γδ T cells between the healthy population and HCC patients. We found that even though the global proportion of γδ T cells in CD3^+^ T cells is not statistically different between healthy and HCC populations, the Vδ1^+^ subset dramatically elevated, while the Vδ2^+^ subset strikingly reduced in the HCC population (p < 0.0001) (**Figure 6E**). This leads to a striking augmentation of the Vδ1^+^/Vδ2^+^ ratio in the HCC population. Further analysis revealed the significant reduction of NKG2D expression in the Vδ1^+^ subset, but not expressions of PD1, NKP30, and NKP46. For the Vδ2^+^ subset, expression of PD1 is greatly increased in the HCC group, implying depressed antitumor immunity of Vδ2^+^ γδ T cells. For other makers, NKG2D, NKP30, and NKP46 show no statistical difference between the healthy and HCC groups (**Figures 6F, G**).

## Discussion

It is commonly recognized that the TME is immunosuppressive, which results from many regulatory factors, including low pH value, hypoxia, nutritional deficiency, metabolic pathways remodeling, inflammation, and others (24, 25). Our focus in this work is to decipher the immune landscape of the HCC TME, facilitating clinical efficacy predictions of immunotherapies. Firstly, our global evaluation of HCC transcriptomic data unequivocally reveals that compared with normal tissue, HCC tissue expresses significantly higher inhibitory checkpoint molecules, including CTLA4, HAVCR2, LAG3, PDCD1, PDCD1LG2, TIGIT, and SIGLEC15. Notably, expressions of these checkpoint molecules are independent of the pTNM stages of patients, therefore clearly suggesting that HCC patients with various pTNM stages will have similar responses to ICB therapy. Then, CIBERSORT evaluation revealed that HCC TME is indeed immunosuppressive, evident by significantly more infiltration of Tregs, activated mast cells, and M0 macrophages. Meanwhile, HCC TME strikingly suppresses γδ T-cell infiltration, inhibits the differentiation of macrophages from M0 to M2, dampens the activation of myeloid DCs, and reduces neutrophil infiltration. Importantly, the infiltration of these immune cells shows no statistical difference among samples of different pTNM stages I–IV. Together, we can conclude that HCC TME is highly immunosuppressive, and the patients with different pTNM stages I–IV probably respond to ICB and immune cell therapy similarly.

Inflammation, which causes various types of diseases, is one of the major inducers that closely coordinates with the establishment of the immunosuppressive TME. In HCC TME, inflammation mainly originates from continuous cell death in the context of excessive proliferation of cancer cells. Cancer cell death could be classified into two types, immunogenic and non-immunogenic deaths. Ferroptosis and pyroptosis belong to immunogenic cell death, while apoptosis is a type of non-immunogenic cell death. We thus tried to understand how these three types of PCD (apoptosis, ferroptosis, and pyroptosis) are involved in constructing the immunosuppressive HCC TME. We found that in HCC, most marker genes of the three types of “-optosis” are significantly upregulated, implicating that apoptosis, ferroptosis, and pyroptosis indeed occur routinely in HCC TME. Notably, further inspections indicated that only 7 apoptosis marker genes, 5 ferroptosis marker genes, and 3 pyroptosis marker genes have strong correlations (p < 0.01) with the OS rate. This implies that the occurrence of “-optosis” in HCC TME can directly impact the clinical prognosis. Moreover, most of these 15 marker genes are upregulated gradually from pTNM stage I to IV, implying an increasing tendency of “-optosis” from the early to the late stage of liver cancer.

Previously, the gene–gene correlations between checkpoint molecules (both stimulatory and inhibitory) and “-optosis” related marker genes remain largely unknown. After thorough analysis, we found that nearly all “-optosis” marker genes (p < 0.01 correlation with the OS) are positively and significantly correlated with expressions of checkpoint molecules except BCAP31, RPL8, and NLRP6 (**Figures 2E**, **3E**, and **4D**). This partially explains why the higher expression of “-optosis” marker genes leads to poor prognoses. Even though these marker genes positively correlated with infiltrations of immune cells (B cells, macrophages, myeloid DCs, neutrophils, CD4^+^ T, and CD8^+^ T) as well (**Figures 2F**, **3F**, and **4E**), further immune cell subsets explorations revealed that the enriched immune cells mainly consist of inhibitory cells (Tregs and activated mast cells) and resting immune cells (resting macrophages and DCs) (**Figure 5D**). Meanwhile, antitumor effector immune cells are greatly suppressed, including γδ T cells, monocytes, and neutrophils. Such immune phenotype of HCC TME probably links with checkpoint molecules CD27, CD40, CD28, and CTLA4, which leads to enriched inhibitory cells while depleting effector immune cells, although this hypothesis needs to be validated experimentally. These pieces of evidence altogether indicated that the three types of “-optosis” collaboratively orchestrated the immunosuppressive nature of HCC TME, thus addressing why HCC patients with higher expression of “-optosis” marker genes had poorer responses to ICB therapy.

Since γδ T cells reside preferentially in the liver and play a key role in preventing liver tumorigenesis, we thus analyzed phenotypic profiles of circulating γδ T cells in HCC and healthy populations. Universally, Vδ2^+^ is dominant over Vδ1^+^ subset in the peripheral blood of the healthy population and accounts for over 50% of total γδ T cells. Under immune-suppressed conditions, the Vδ2^+^ subset is slowly depleted in the context of long-term stimulation of phosphoantigens presented by cancer cells and myeloid cells; however, the Vδ1^+^ subset can survive, as it is more Treg-like. This interpretation can be supported by results that the Vδ1^+^ subset was enriched in HCC patient blood, while the percentage of the Vδ2^+^ subset was strikingly decreased. Because the data show that more than 90% of the healthy population has the Vδ1^+^/Vδ2^+^ ratio ≤ 4 and more than 63.4% of HCC patients have the Vδ1^+^/Vδ2^+^ ratio > 4, we thus propose that 4 can be used as the threshold value of the Vδ1^+^/Vδ2^+^ ratio since higher Vδ1^+^/Vδ2^+^ ratio implies more suppressive immunity of the host. Whether or not the proposed Vδ1^+^/Vδ2^+^ ratio can be a predictive factor for discriminating healthy (≤4) or sub-healthy conditions (>4 with no medical abnormalities) and for predicting HCC prognosis (≤4 links with better prognosis) needs further validation with a larger scale of clinical samples. Nevertheless, the imbalanced Vδ1^+^/Vδ2^+^ ratio has the potential to be used as an indicator in health checkups and clinical prognosis. Additionally, reduced NKG2D expression of the Vδ1^+^ population suggests depressed cell activation and probably enhanced survival ability of the Vδ1^+^ subset in HCC. In contrast, a significantly higher PD1 expression in the Vδ2^+^ population indicated the depressed cytotoxicity of the Vδ2^+^ γδ T cell in the HCC group (**Figures 6F, G**). Together, we can conclude that in the context of HCC, the Vδ1^+^ subset survives and consists of the main γδ T-cell population, whereas the Vδ2^+^ subset is more vulnerable to long-term stimulation of phosphoantigens and is easily depleted or suppressed.

**Figure 7:**
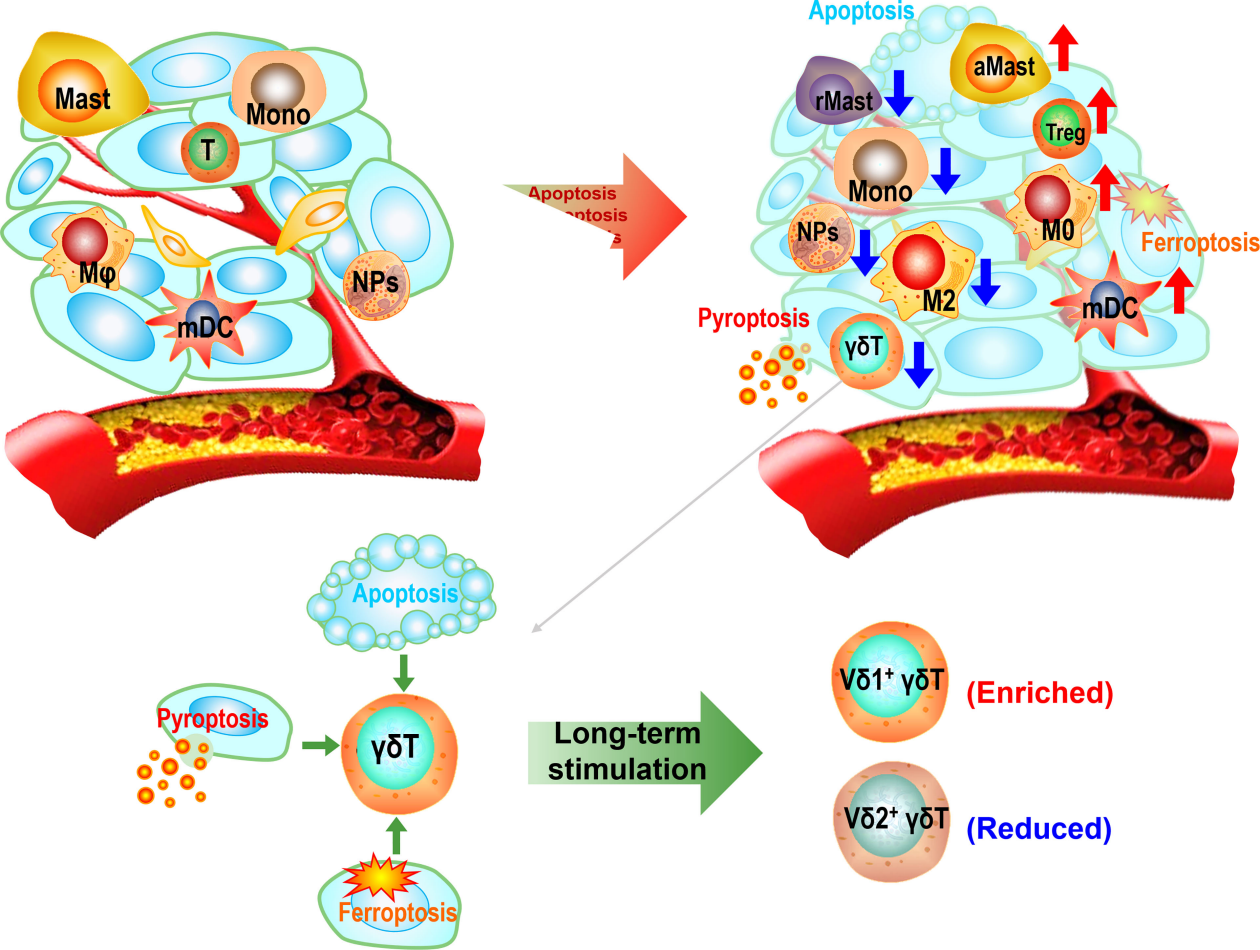
The sketch diagram depicts the establishment of immunosuppressive hepatocellular carcinoma (HCC) tumor microenvironment (TME). The excessive growth of cancer cells accompanies by apoptotic, ferroptotic, and pyroptotic deaths, leading to the construction of the inflammatory microenvironment and subsequently immunosuppressive TME. The immune profile of the TME appears as enrichments of Tregs, activated mast cells, M0, and resting myeloid dendritic cells (DCs) but reductions of γδ T cells, M2 cells, monocytes, resting mast cells, and neutrophils. Long-term stimulation in the TME leads to a predominant Vδ1^+^ population in total γδ T cells.

In conclusion, the HCC TME is highly immunosuppressive, leading to high expressions of inhibitory checkpoint molecules and limited infiltrations of antitumor immune cells. Our study revealed that apoptosis, ferroptosis, and pyroptosis conspiringly induce the establishment of the immunosuppressive HCC TME. The immunosuppressive landscape of the HCC TME was shaped by the high expression of inhibitory checkpoint molecules and enrichments of Tregs, activated mast cells, M0, and non-activated myeloid DCs but low enrichments of antitumor effector cells such as γδ T cells, M2 cells, monocytes, and neutrophils (**Figure 7**). Our bioinformatics evidence also showed that the inactivation of the TME infiltrating immune cells might be mediated by checkpoint molecules. Finally, since cytotoxic Vδ2^+^ γδ T cell is selectively depleted whereas Treg-like Vδ1^+^ γδ T cell is upregulated in HCC, the adoptive transfer of allogeneic Vδ2^+^ γδ T cells could be a promising immunotherapeutic strategy for this malignant cancer (4, 5).

## Data Availability Statement

The datasets presented in this study can be found in online repositories. The names of the repository/repositories and accession number(s) can be found in the article/**Supplementary material**.

## Author Contributions

Work supervision and project design: YH and YW. Experiments: DC, MH, JL, and YJL. Data analysis and bioinformatics: YW and YH. Data discussion: YW, YH, JH, LL, and ZY. Manuscript drafting, proofreading, and revision: YH and YW. All authors approved manuscript submission.

## Conflict of Interest

ZY is the founder of the Shuangzhi Purui Medical Laboratory Co., Ltd. (Wuhan, China).

The remaining authors declare that the research was conducted in the absence of any commercial or financial relationships that could be construed as a potential conflict of interest.

## Publisher’s Note

All claims expressed in this article are solely those of the authors and do not necessarily represent those of their affiliated organizations, or those of the publisher, the editors and the reviewers. Any product that may be evaluated in this article, or claim that may be made by its manufacturer, is not guaranteed or endorsed by the publisher.
