# Supplementary material for: Apoptosis, Pyroptosis, and Ferroptosis Conspiringly Induce Immunosuppressive Hepatocellular Carcinoma Microenvironment and γδ T-Cell Imbalance
Source: Front Immunol. 2022 Apr 4;13:845974. doi: 10.3389/fimmu.2022.845974 (PMC9013882; doi:10.3389/fimmu.2022.845974)
Supplement: Supplementary file 2 [file Presentation_1.pptx]

## Slide 1
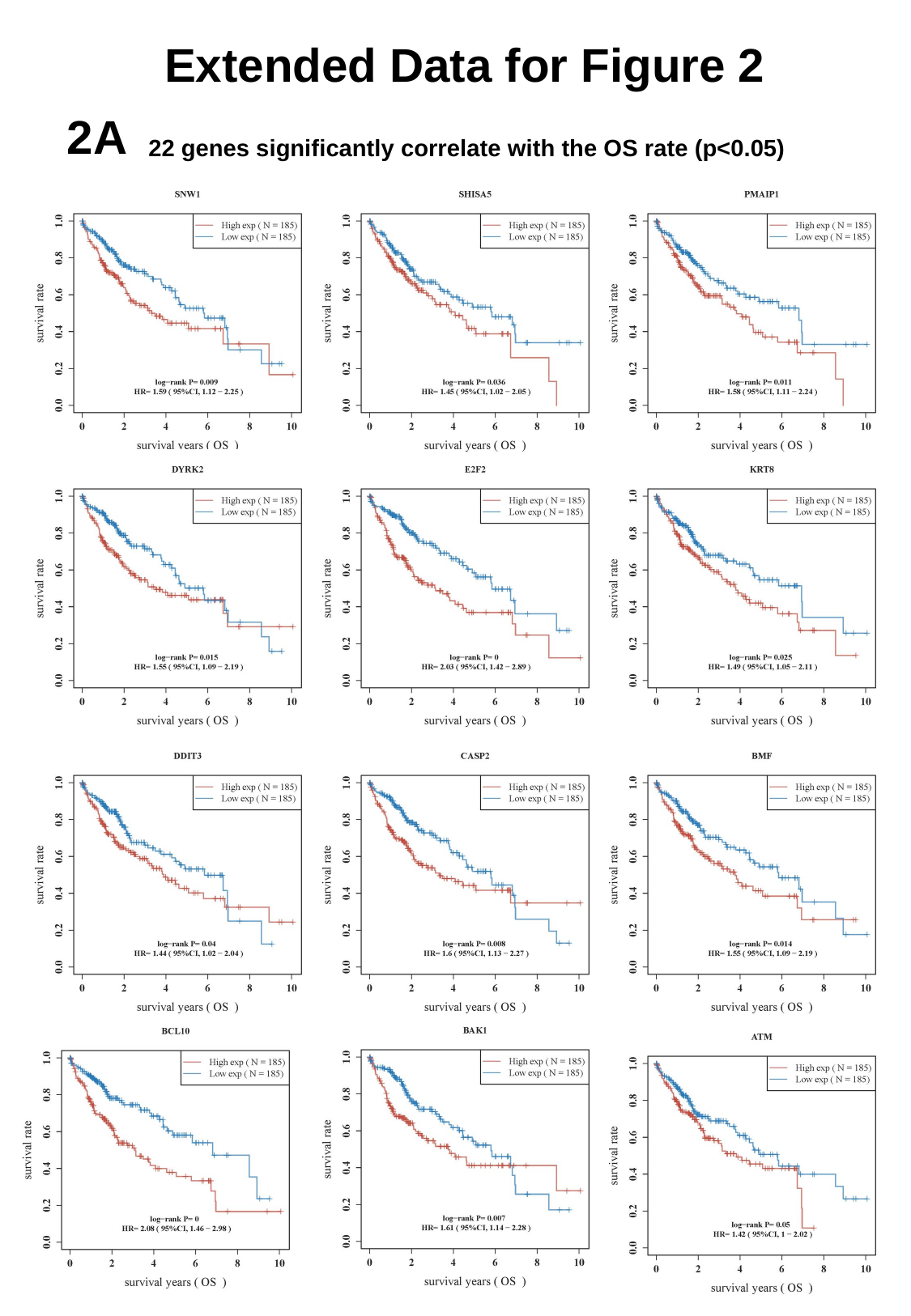

Extended Data for Figure 2
2A
22 genes significantly correlate with the OS rate (p<0.05)

## Slide 2
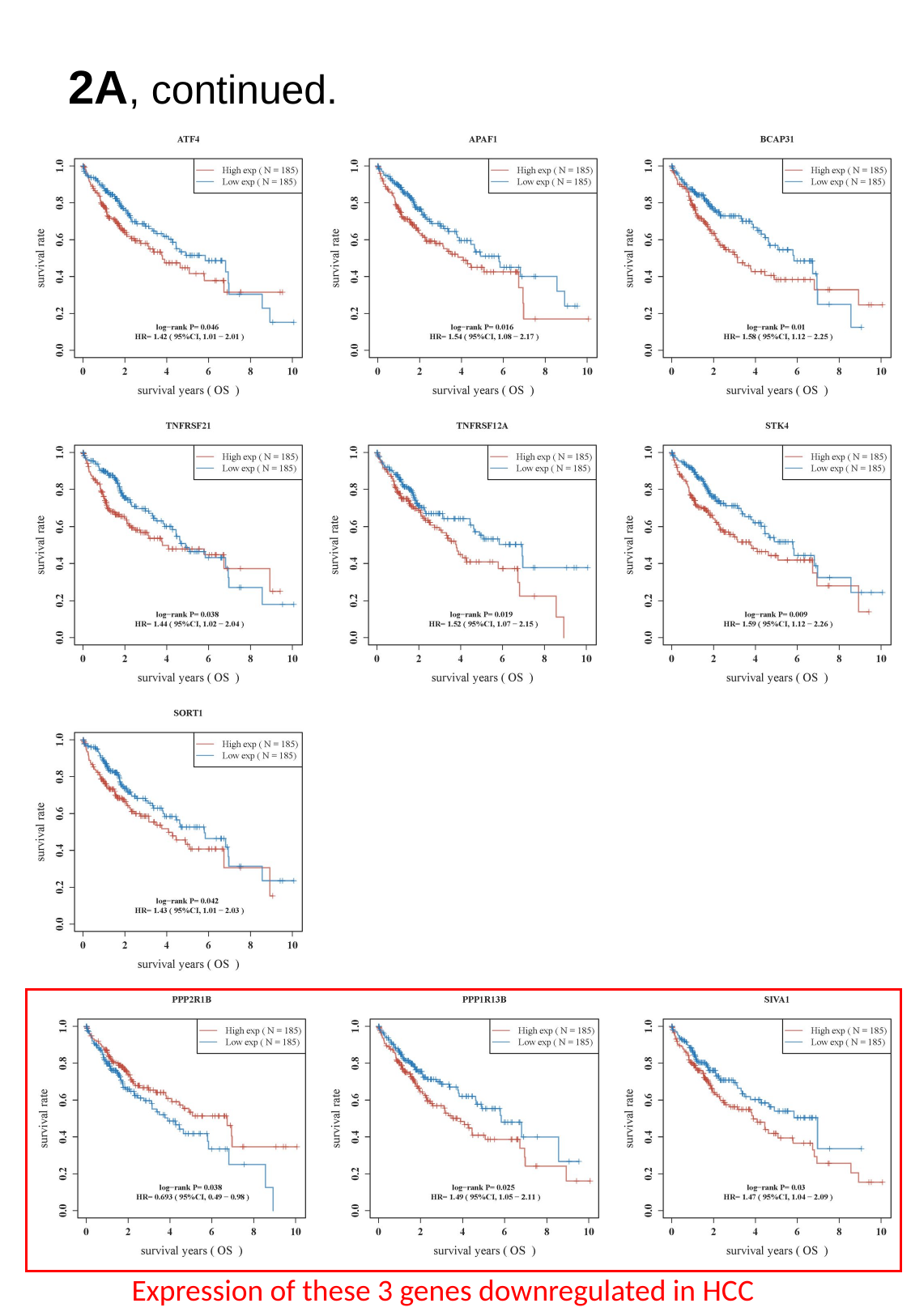

2A, continued.
Expression of these 3 genes downregulated in HCC

## Slide 3
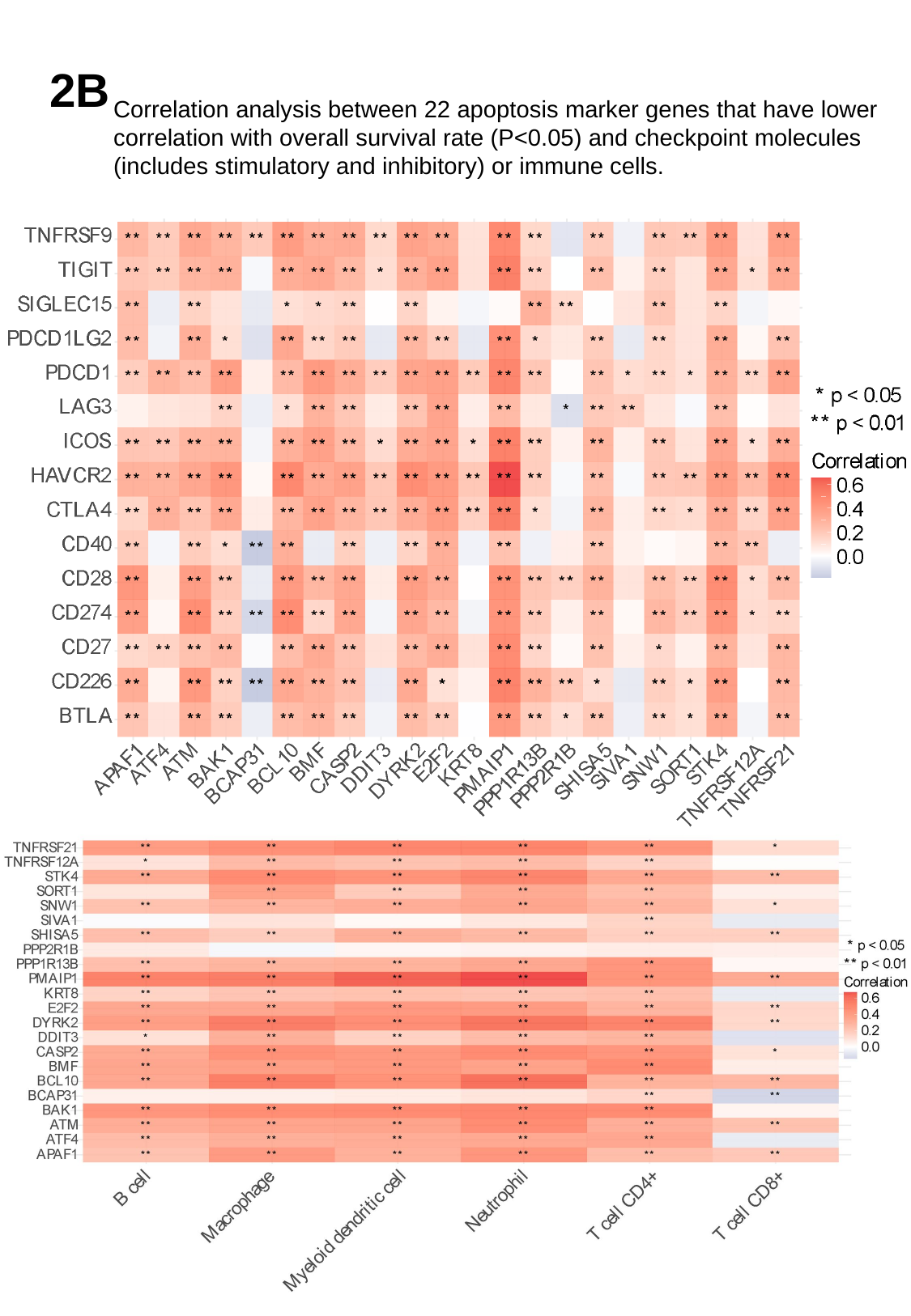

2B
Correlation analysis between 22 apoptosis marker genes that have lower correlation with overall survival rate (P<0.05) and checkpoint molecules (includes stimulatory and inhibitory) or immune cells.

## Slide 4
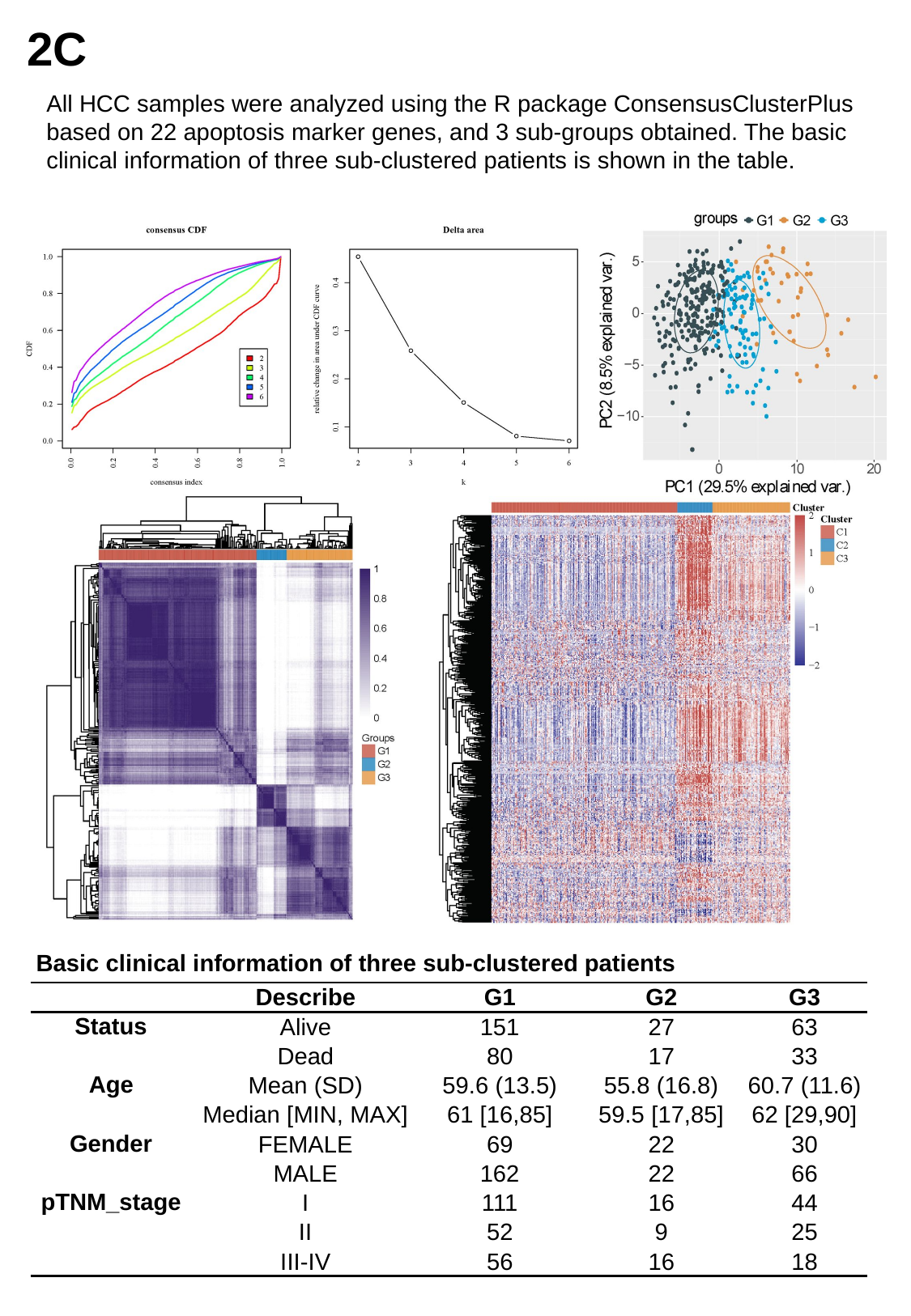

2C
All HCC samples were analyzed using the R package ConsensusClusterPlus based on 22 apoptosis marker genes, and 3 sub-groups obtained. The basic clinical information of three sub-clustered patients is shown in the table.
Basic clinical information of three sub-clustered patients
| | Describe | G1 | G2 | G3 |
| --- | --- | --- | --- | --- |
| Status | Alive | 151 | 27 | 63 |
| | Dead | 80 | 17 | 33 |
| Age | Mean (SD) | 59.6 (13.5) | 55.8 (16.8) | 60.7 (11.6) |
| | Median [MIN, MAX] | 61 [16,85] | 59.5 [17,85] | 62 [29,90] |
| Gender | FEMALE | 69 | 22 | 30 |
| | MALE | 162 | 22 | 66 |
| pTNM\_stage | I | 111 | 16 | 44 |
| | II | 52 | 9 | 25 |
| | III-IV | 56 | 16 | 18 |

## Slide 5
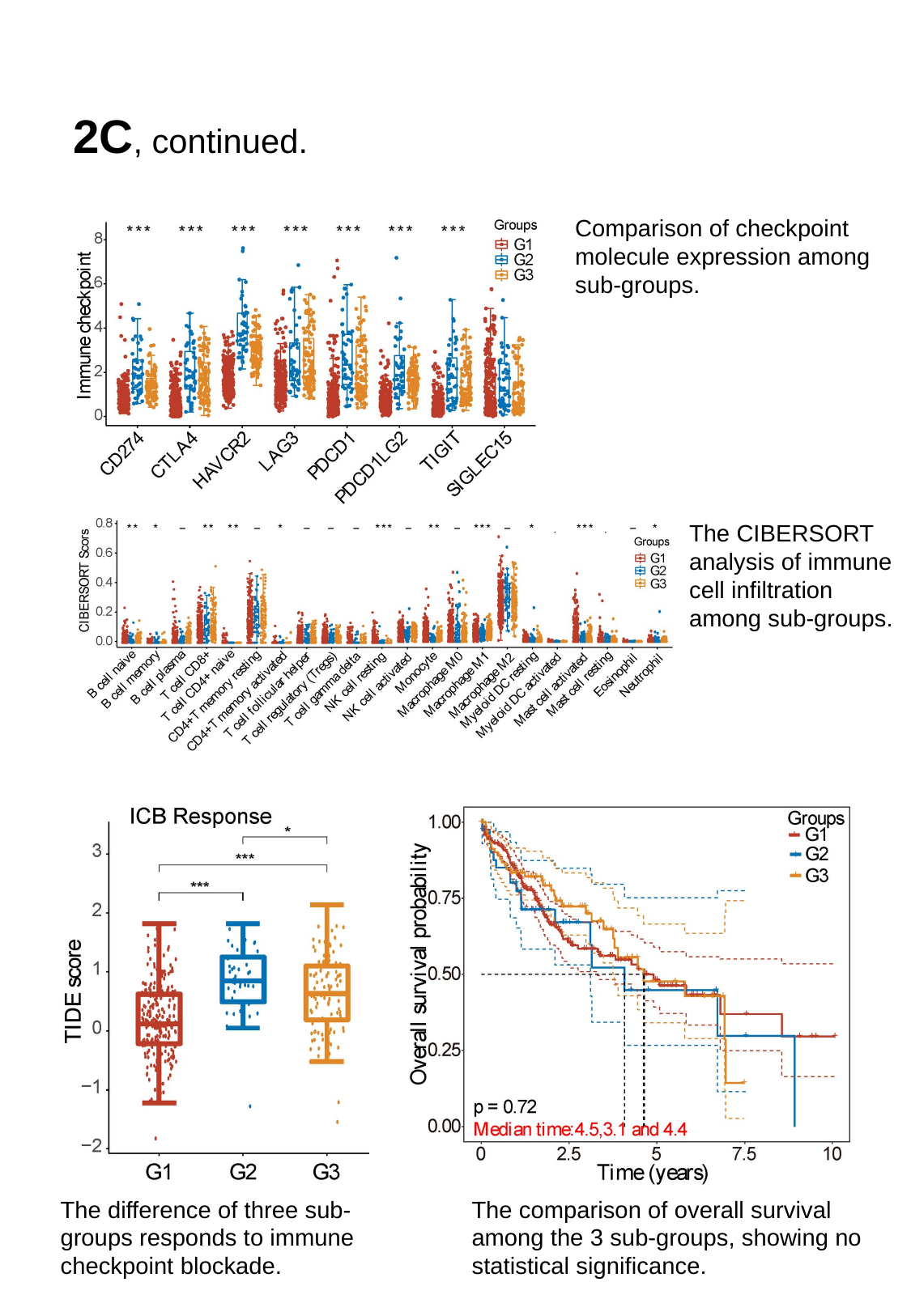

2C, continued.
Comparison of checkpoint molecule expression among sub-groups.
The CIBERSORT analysis of immune cell infiltration among sub-groups.
The difference of three sub-groups responds to immune checkpoint blockade.
The comparison of overall survival among the 3 sub-groups, showing no statistical significance.

## Slide 6
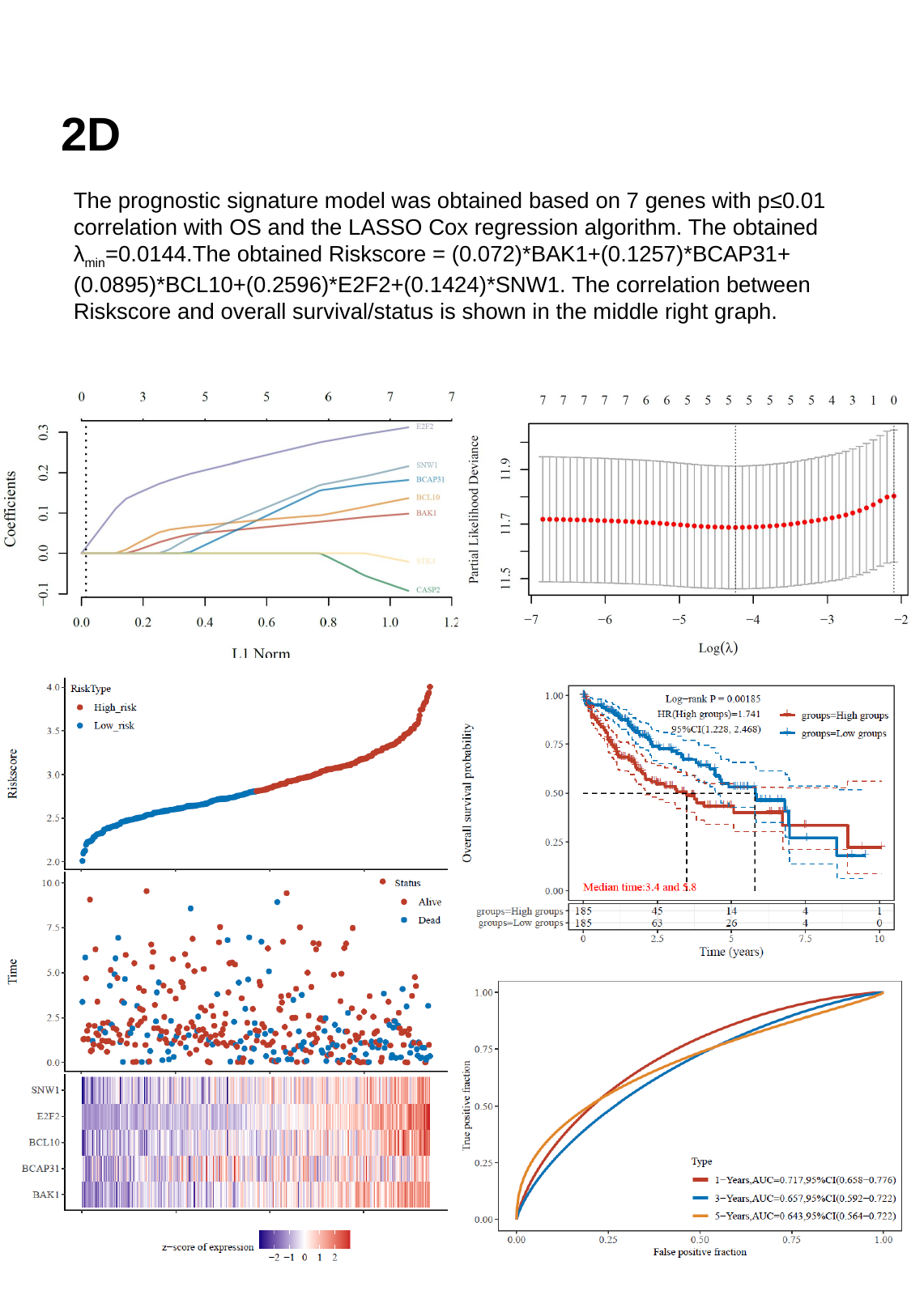

2D
The prognostic signature model was obtained based on 7 genes with p≤0.01 correlation with OS and the LASSO Cox regression algorithm. The obtained λmin=0.0144.The obtained Riskscore = (0.072)*BAK1+(0.1257)*BCAP31+ (0.0895)*BCL10+(0.2596)*E2F2+(0.1424)*SNW1. The correlation between Riskscore and overall survival/status is shown in the middle right graph.

## Slide 7
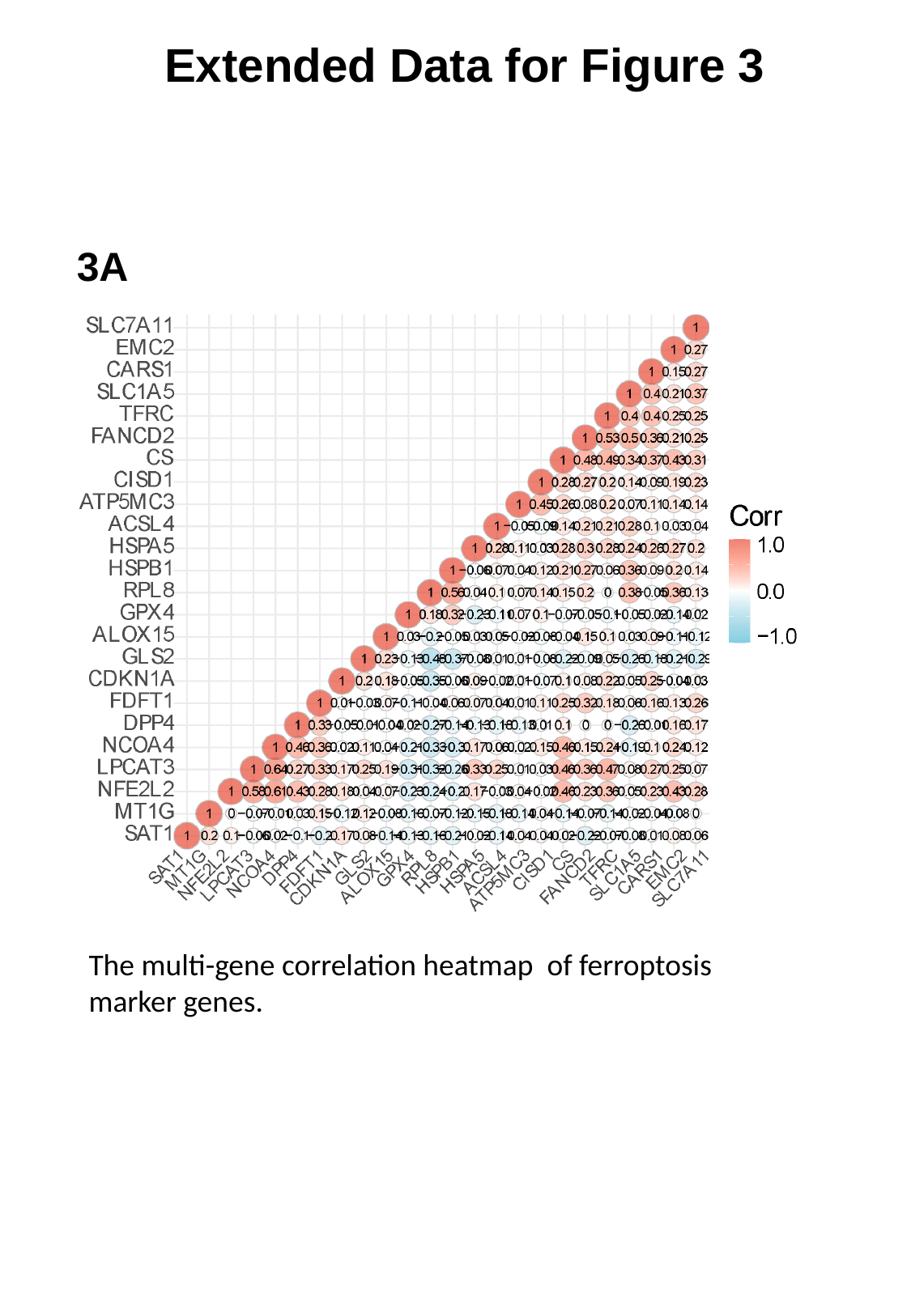

Extended Data for Figure 3
3A
The multi-gene correlation heatmap of ferroptosis marker genes.

## Slide 8
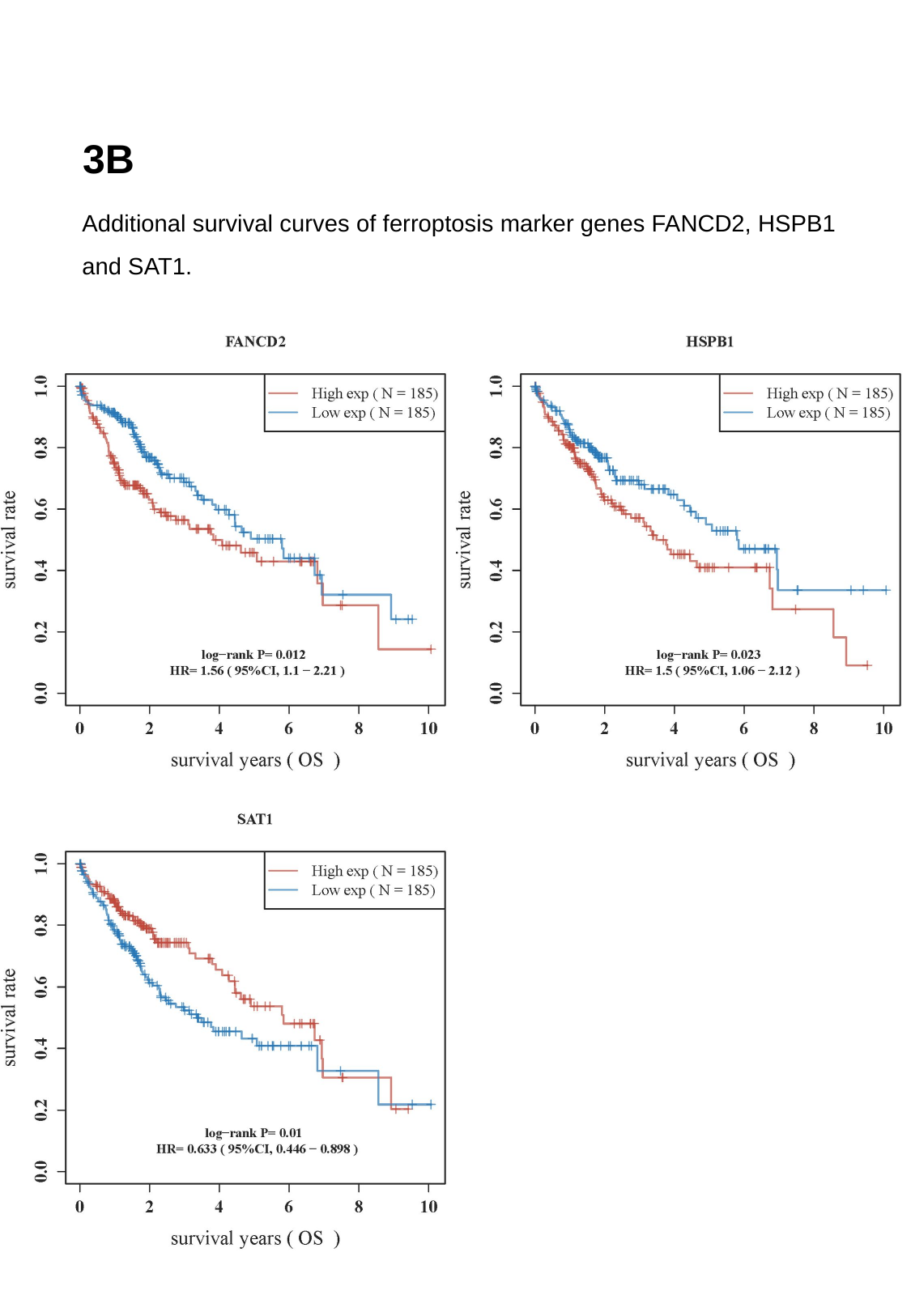

3B
Additional survival curves of ferroptosis marker genes FANCD2, HSPB1 and SAT1.

## Slide 9
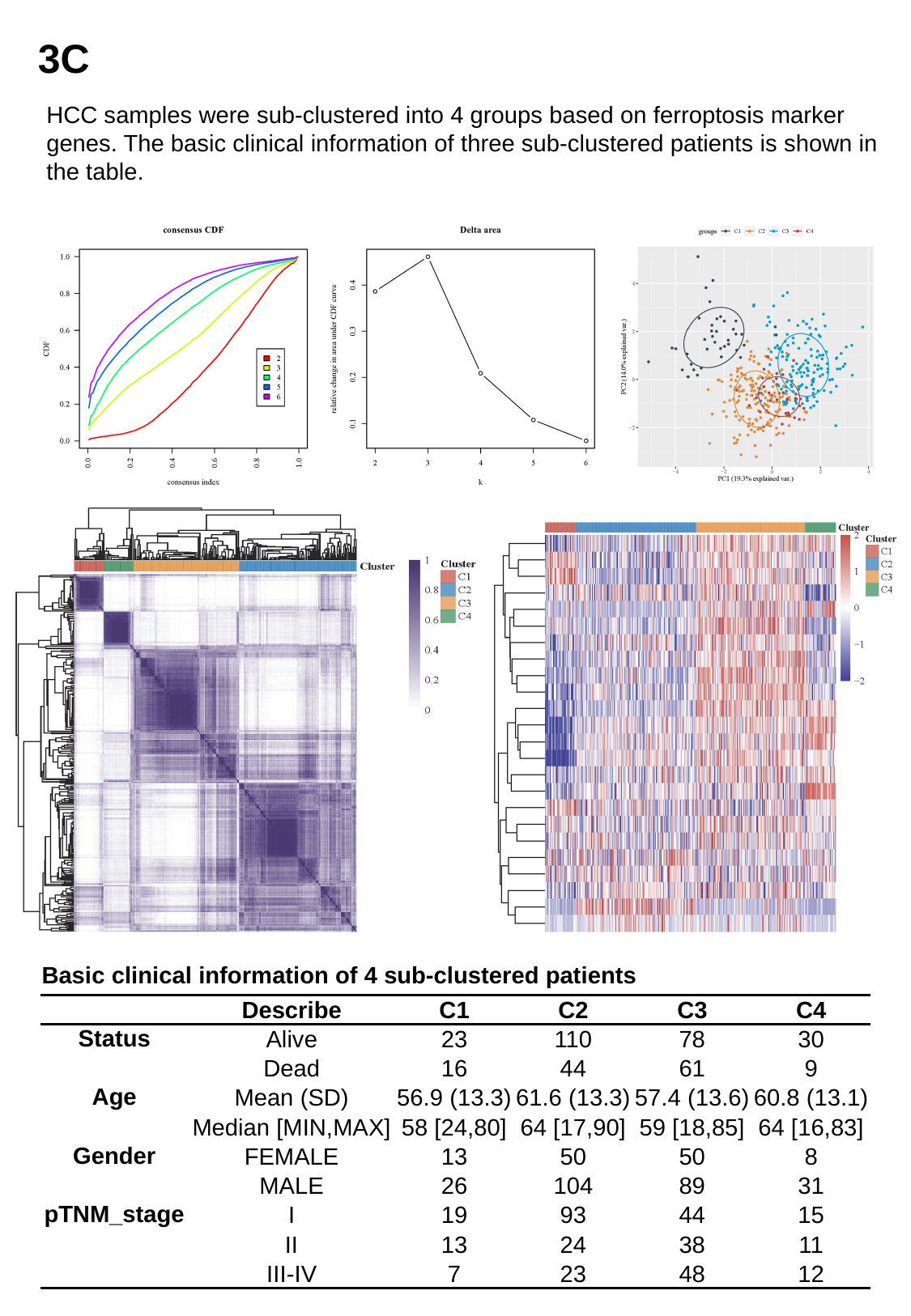

3C
HCC samples were sub-clustered into 4 groups based on ferroptosis marker genes. The basic clinical information of three sub-clustered patients is shown in the table.
Basic clinical information of 4 sub-clustered patients
| | Describe | C1 | C2 | C3 | C4 |
| --- | --- | --- | --- | --- | --- |
| Status | Alive | 23 | 110 | 78 | 30 |
| | Dead | 16 | 44 | 61 | 9 |
| Age | Mean (SD) | 56.9 (13.3) | 61.6 (13.3) | 57.4 (13.6) | 60.8 (13.1) |
| | Median [MIN,MAX] | 58 [24,80] | 64 [17,90] | 59 [18,85] | 64 [16,83] |
| Gender | FEMALE | 13 | 50 | 50 | 8 |
| | MALE | 26 | 104 | 89 | 31 |
| pTNM\_stage | I | 19 | 93 | 44 | 15 |
| | II | 13 | 24 | 38 | 11 |
| | III-IV | 7 | 23 | 48 | 12 |

## Slide 10
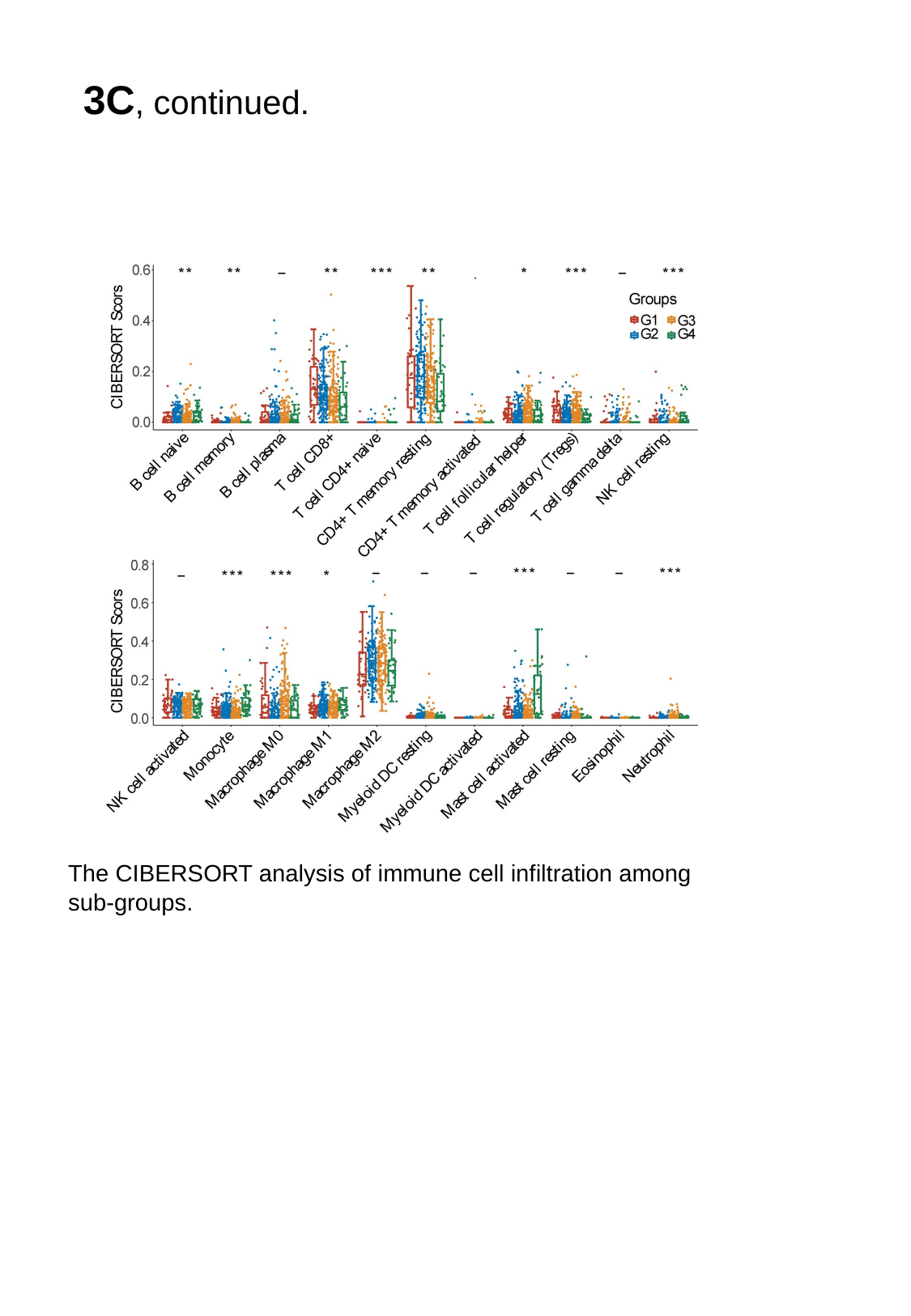

3C, continued.
The CIBERSORT analysis of immune cell infiltration among sub-groups.

## Slide 11
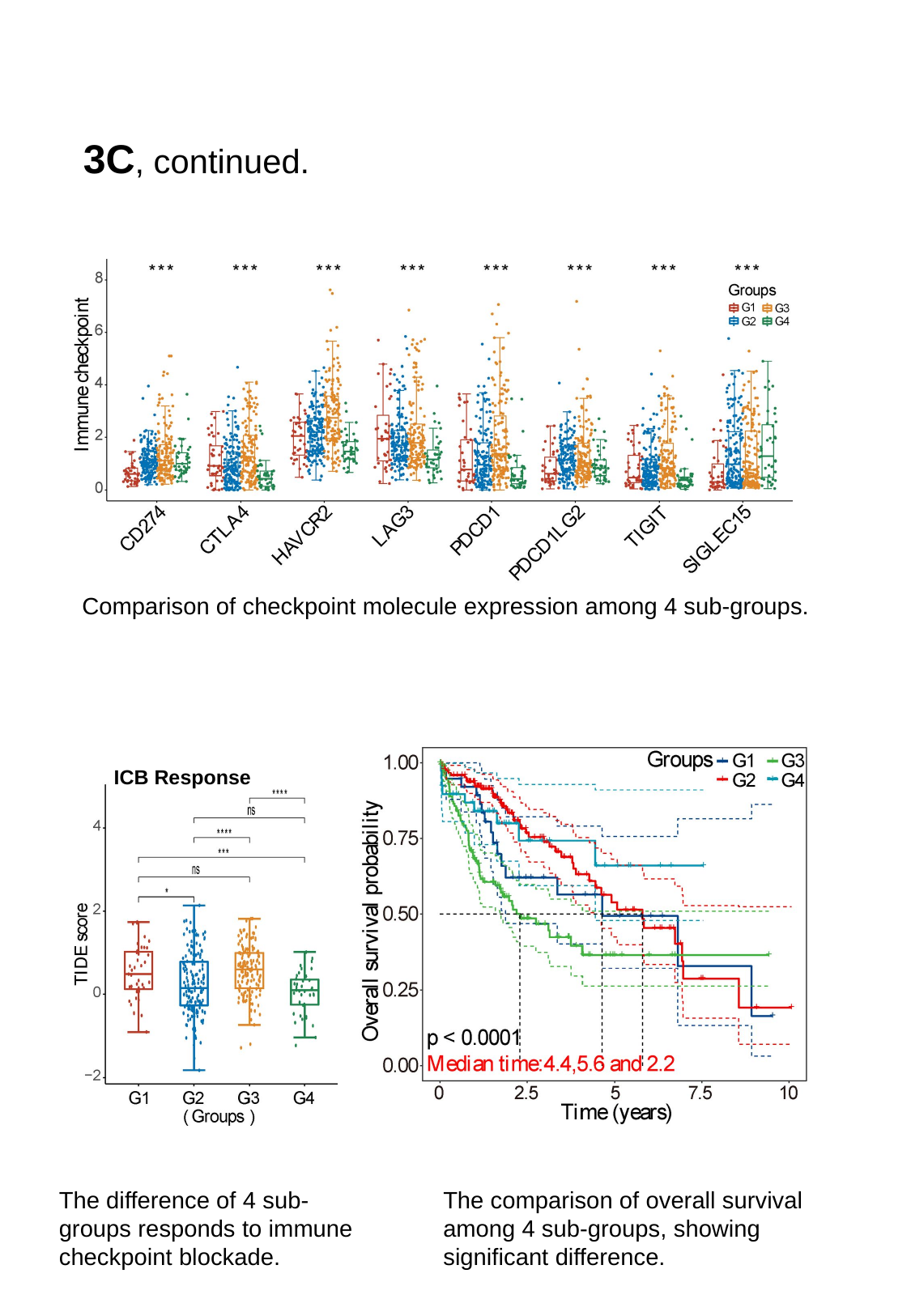

3C, continued.
Comparison of checkpoint molecule expression among 4 sub-groups.
ICB Response
The difference of 4 sub-groups responds to immune checkpoint blockade.
The comparison of overall survival among 4 sub-groups, showing significant difference.

## Slide 12
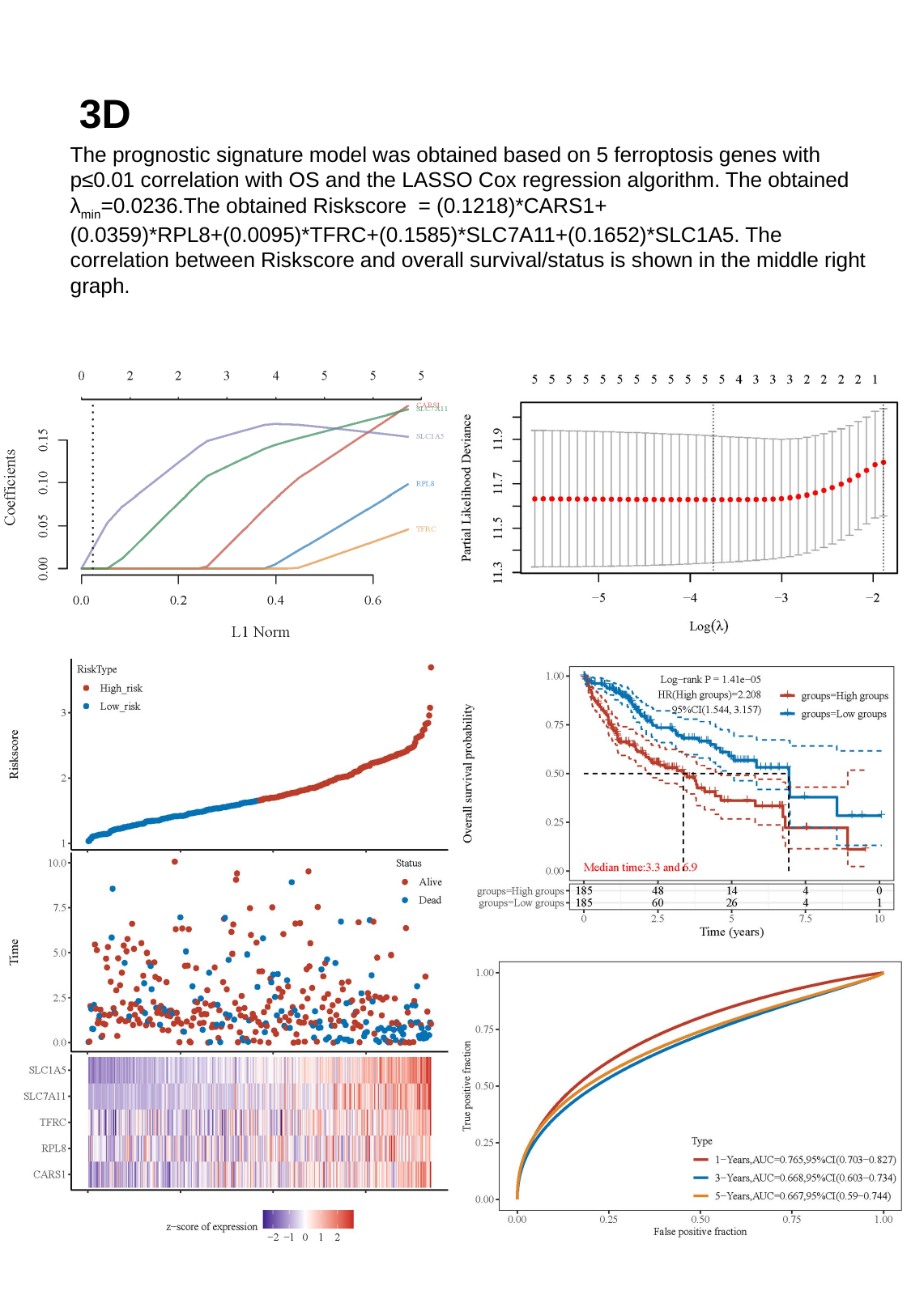

3D
The prognostic signature model was obtained based on 5 ferroptosis genes with p≤0.01 correlation with OS and the LASSO Cox regression algorithm. The obtained λmin=0.0236.The obtained Riskscore = (0.1218)*CARS1+ (0.0359)*RPL8+(0.0095)*TFRC+(0.1585)*SLC7A11+(0.1652)*SLC1A5. The correlation between Riskscore and overall survival/status is shown in the middle right graph.

## Slide 13
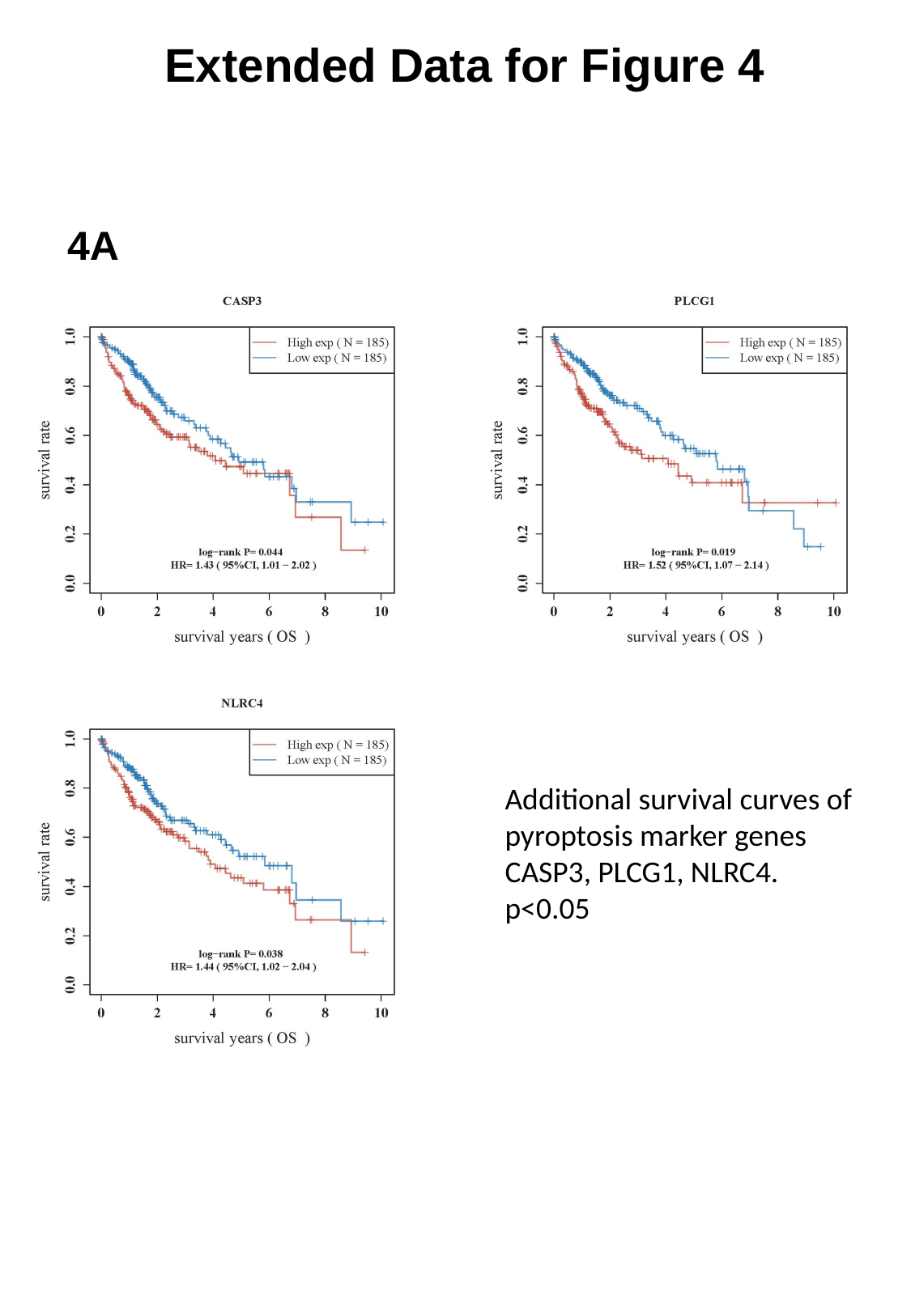

Extended Data for Figure 4
4A
Additional survival curves of pyroptosis marker genes CASP3, PLCG1, NLRC4. p<0.05

## Slide 14
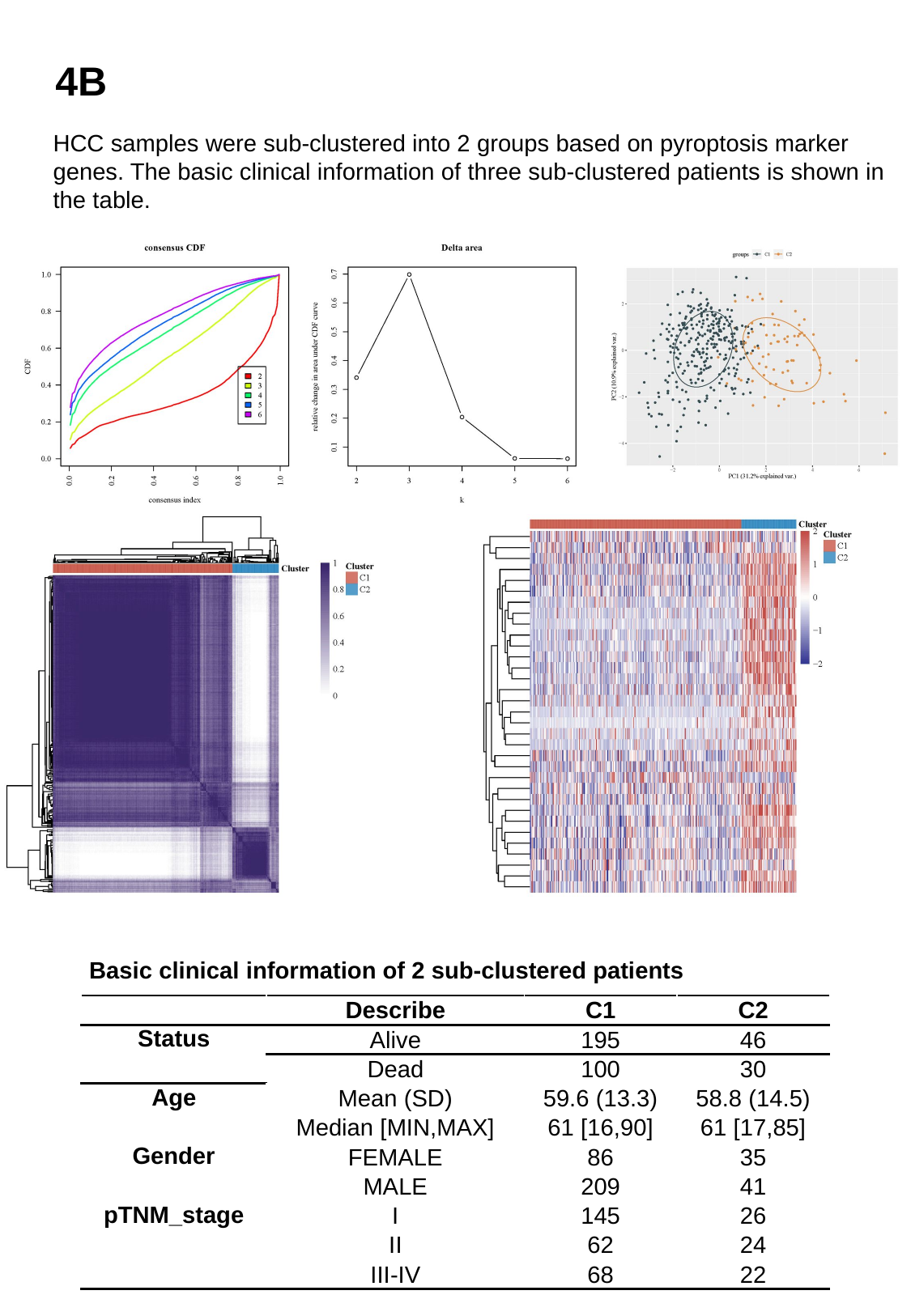

4B
HCC samples were sub-clustered into 2 groups based on pyroptosis marker genes. The basic clinical information of three sub-clustered patients is shown in the table.
Basic clinical information of 2 sub-clustered patients
| | Describe | C1 | C2 |
| --- | --- | --- | --- |
| Status | Alive | 195 | 46 |
| | Dead | 100 | 30 |
| Age | Mean (SD) | 59.6 (13.3) | 58.8 (14.5) |
| | Median [MIN,MAX] | 61 [16,90] | 61 [17,85] |
| Gender | FEMALE | 86 | 35 |
| | MALE | 209 | 41 |
| pTNM\_stage | I | 145 | 26 |
| | II | 62 | 24 |
| | III-IV | 68 | 22 |

## Slide 15
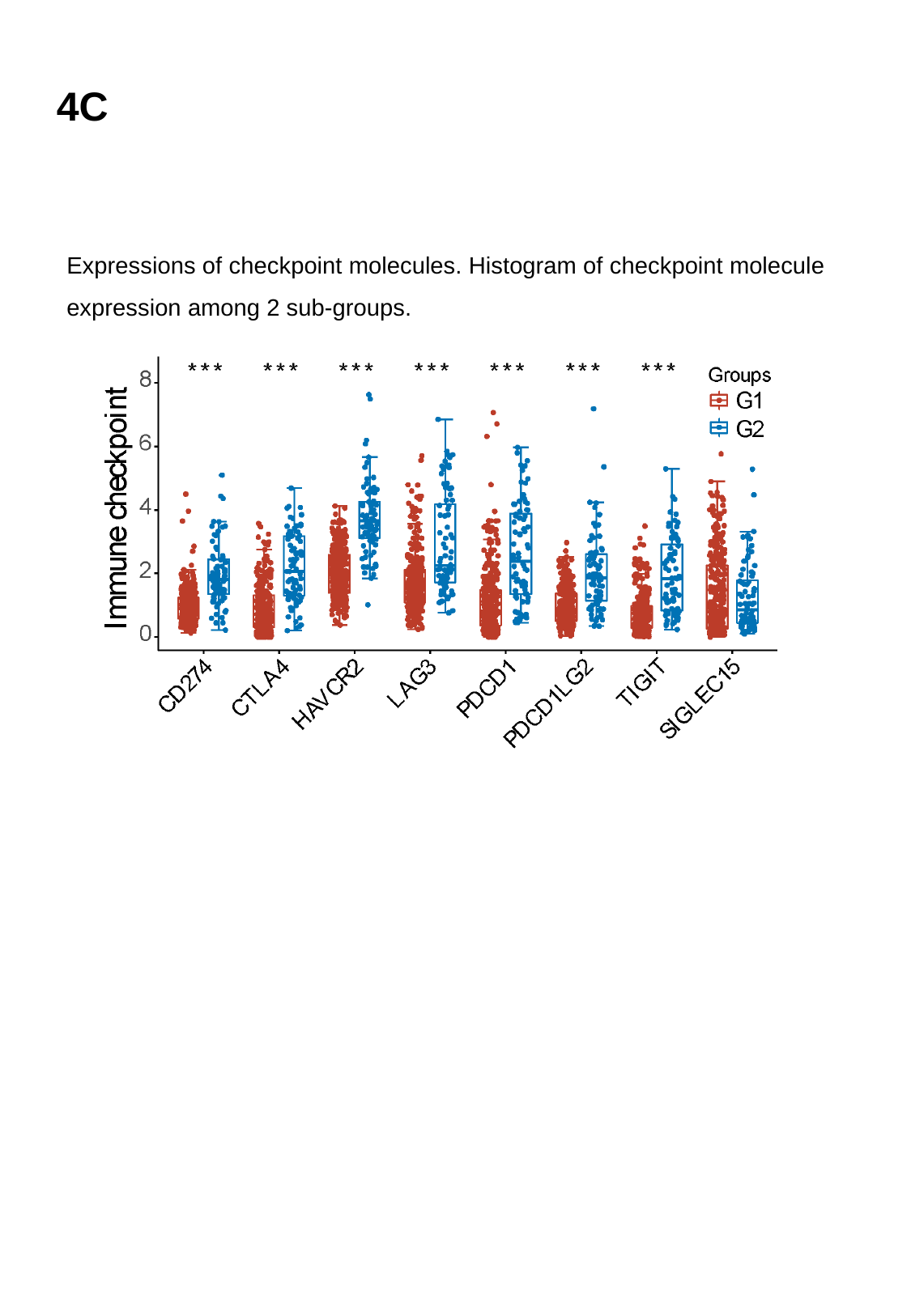

4C
Expressions of checkpoint molecules. Histogram of checkpoint molecule expression among 2 sub-groups.

## Slide 16
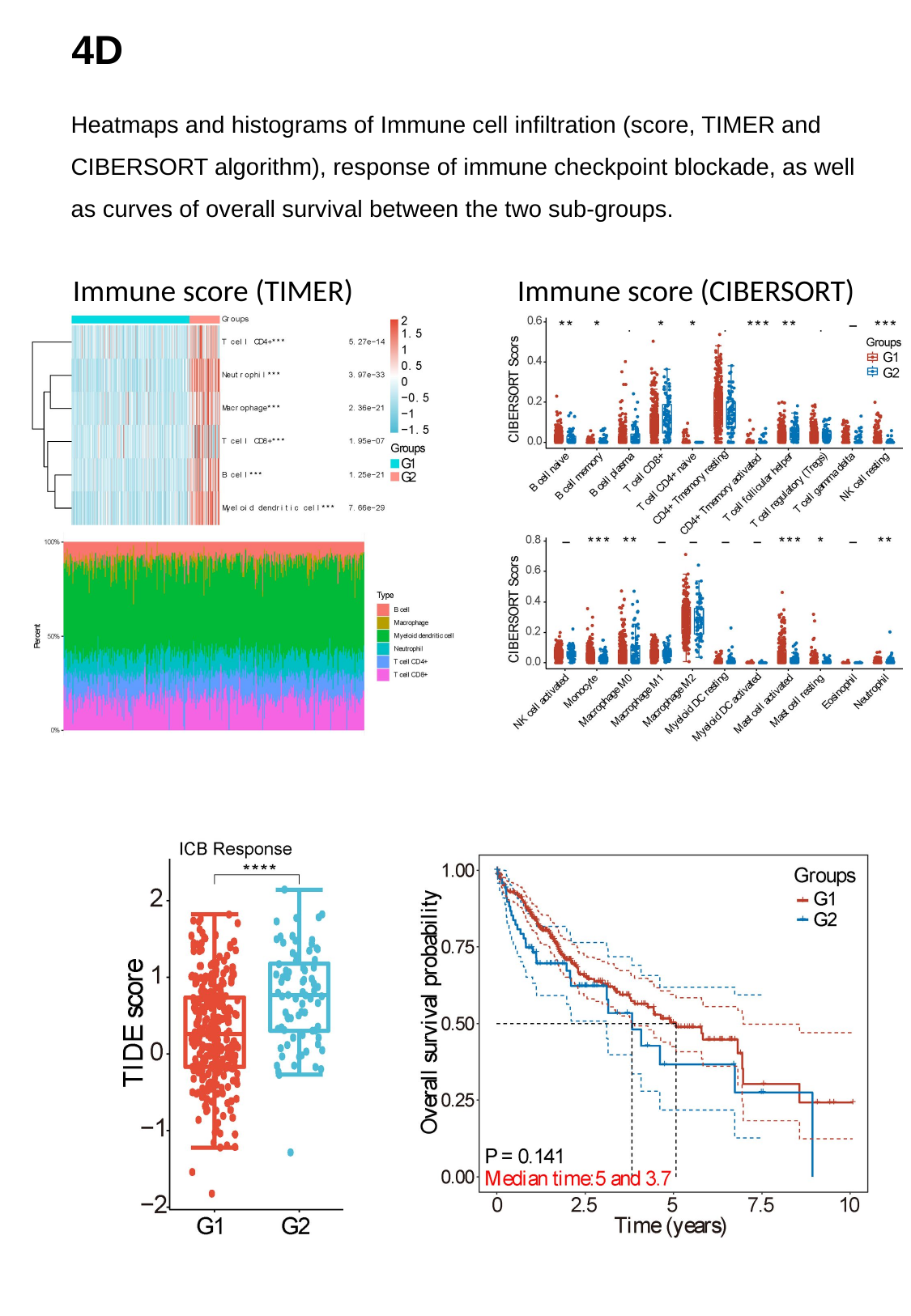

4D
Heatmaps and histograms of Immune cell infiltration (score, TIMER and CIBERSORT algorithm), response of immune checkpoint blockade, as well as curves of overall survival between the two sub-groups.
Immune score (CIBERSORT)
Immune score (TIMER)

## Slide 17
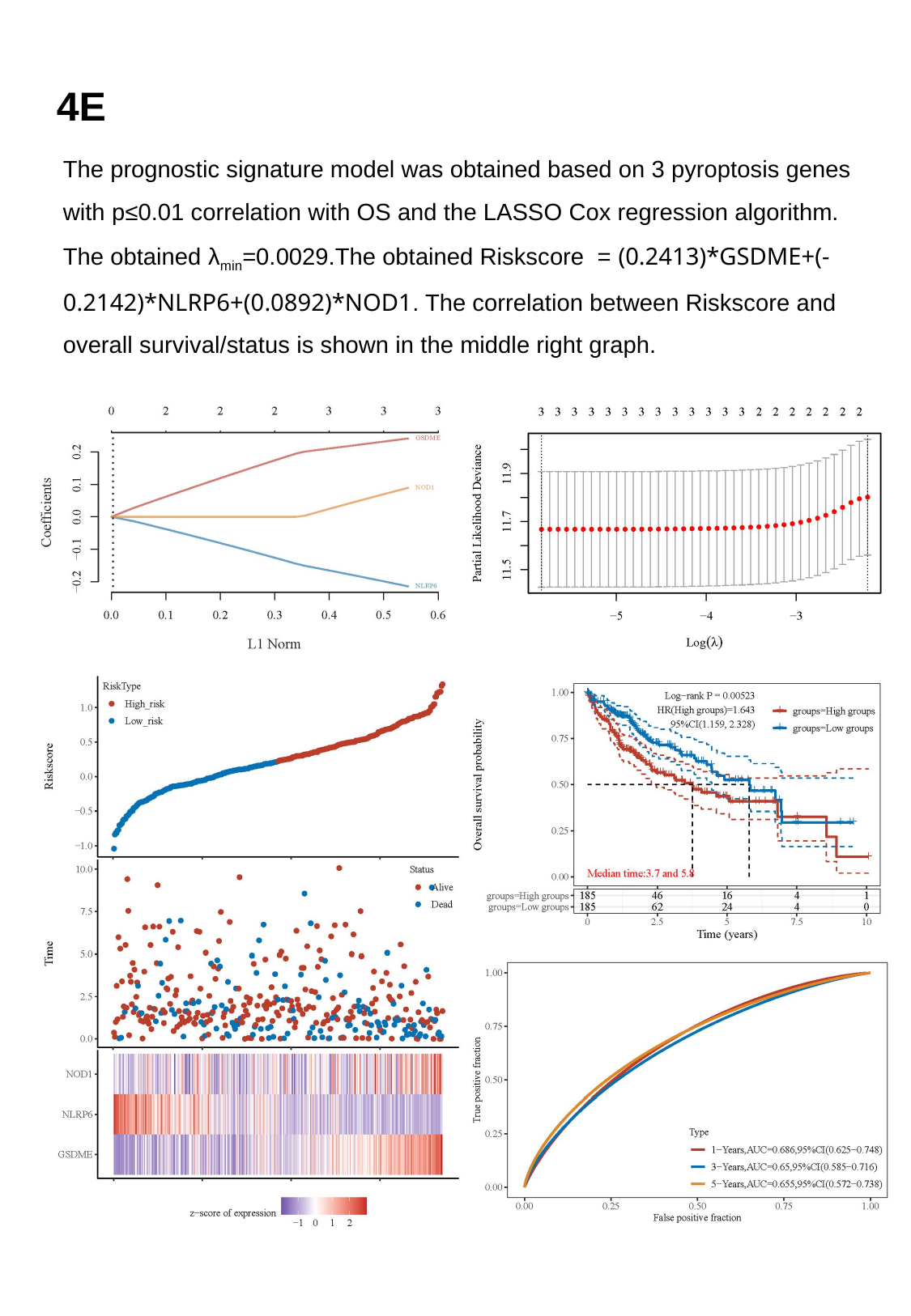

4E
The prognostic signature model was obtained based on 3 pyroptosis genes with p≤0.01 correlation with OS and the LASSO Cox regression algorithm. The obtained λmin=0.0029.The obtained Riskscore = (0.2413)*GSDME+(-0.2142)*NLRP6+(0.0892)*NOD1. The correlation between Riskscore and overall survival/status is shown in the middle right graph.

## Slide 18
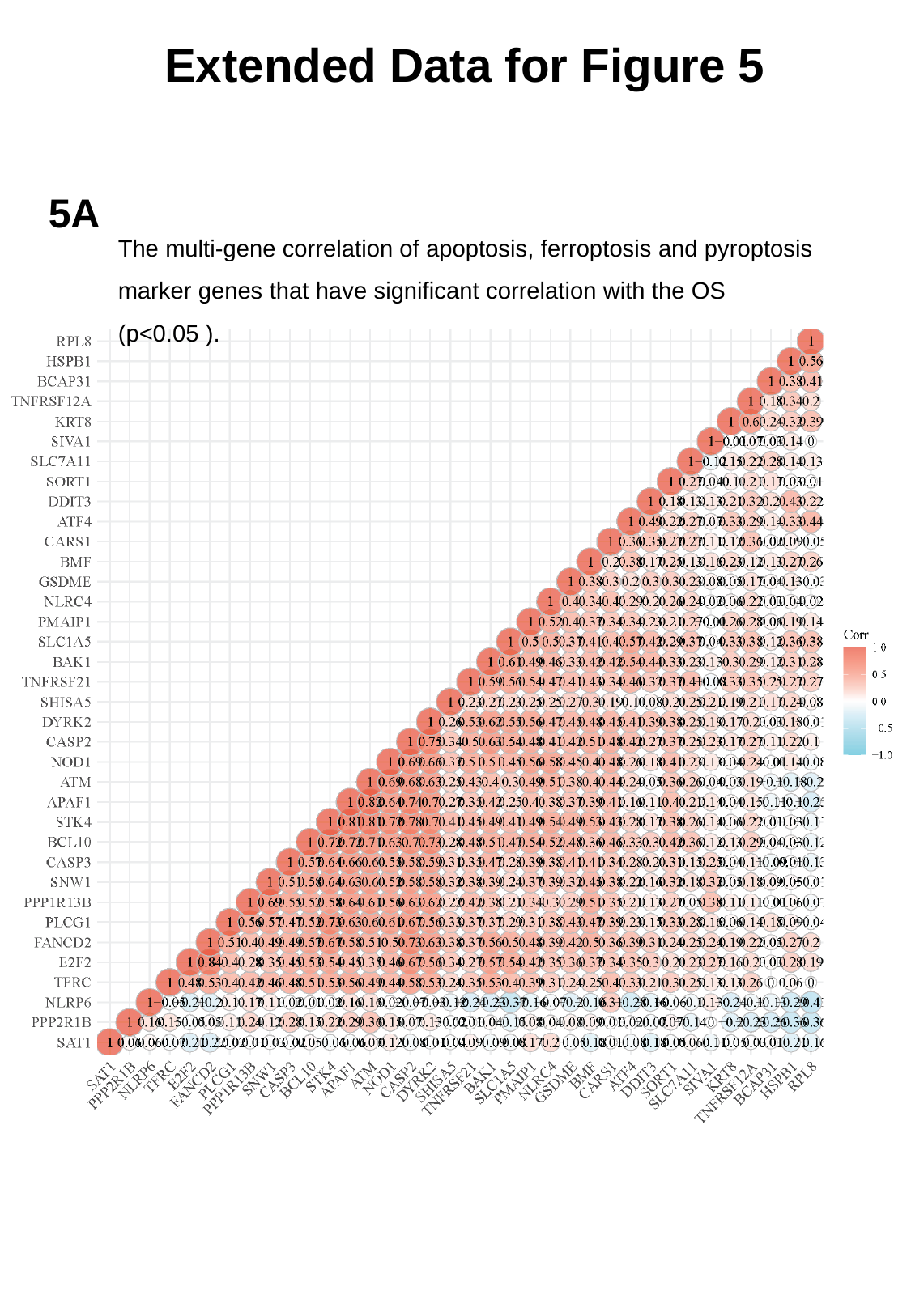

Extended Data for Figure 5
5A
The multi-gene correlation of apoptosis, ferroptosis and pyroptosis marker genes that have significant correlation with the OS (p<0.05 ).

## Slide 19
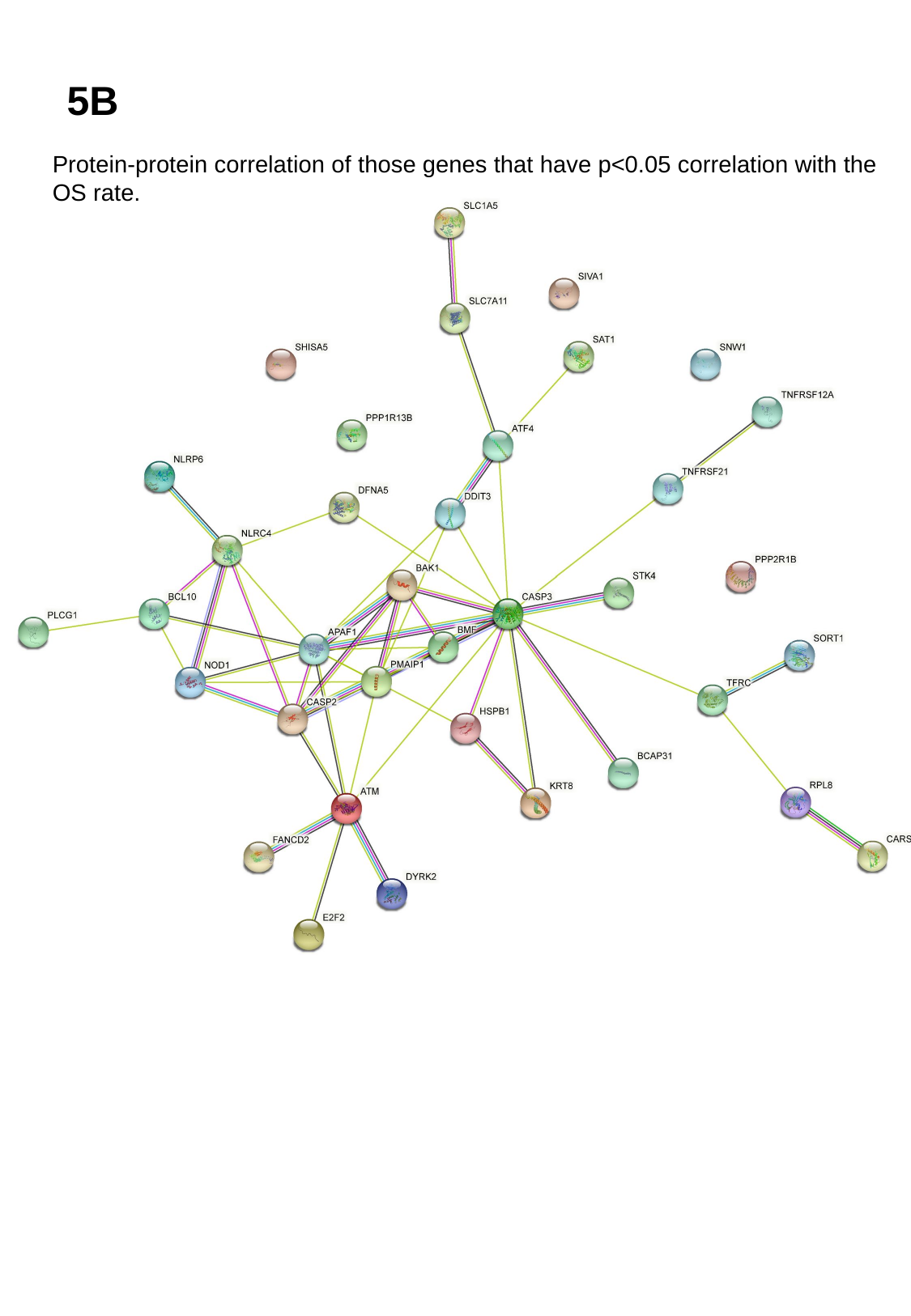

5B
Protein-protein correlation of those genes that have p<0.05 correlation with the OS rate.

## Slide 20
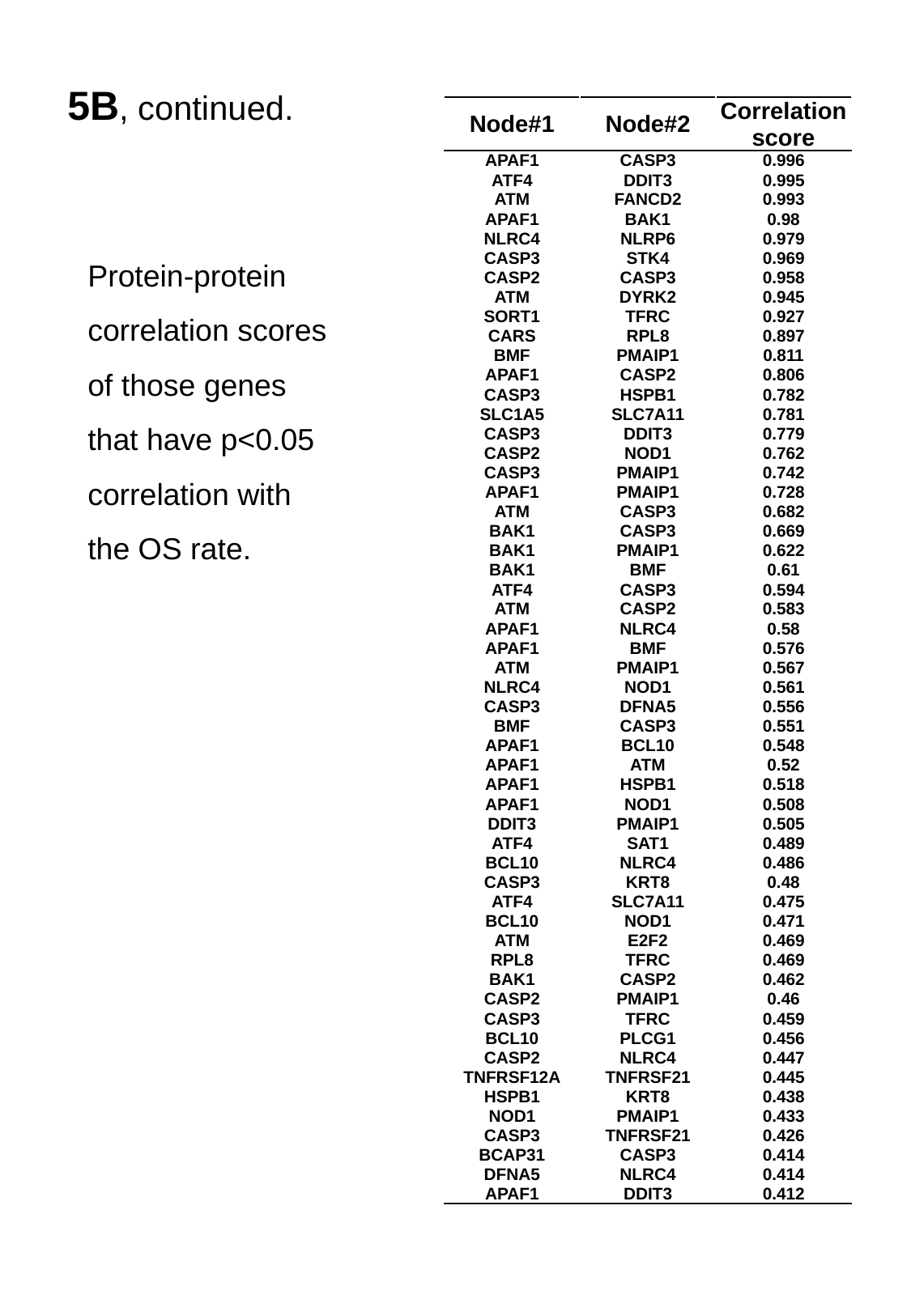

5B, continued.
| Node#1 | Node#2 | Correlation score |
| --- | --- | --- |
| APAF1 | CASP3 | 0.996 |
| ATF4 | DDIT3 | 0.995 |
| ATM | FANCD2 | 0.993 |
| APAF1 | BAK1 | 0.98 |
| NLRC4 | NLRP6 | 0.979 |
| CASP3 | STK4 | 0.969 |
| CASP2 | CASP3 | 0.958 |
| ATM | DYRK2 | 0.945 |
| SORT1 | TFRC | 0.927 |
| CARS | RPL8 | 0.897 |
| BMF | PMAIP1 | 0.811 |
| APAF1 | CASP2 | 0.806 |
| CASP3 | HSPB1 | 0.782 |
| SLC1A5 | SLC7A11 | 0.781 |
| CASP3 | DDIT3 | 0.779 |
| CASP2 | NOD1 | 0.762 |
| CASP3 | PMAIP1 | 0.742 |
| APAF1 | PMAIP1 | 0.728 |
| ATM | CASP3 | 0.682 |
| BAK1 | CASP3 | 0.669 |
| BAK1 | PMAIP1 | 0.622 |
| BAK1 | BMF | 0.61 |
| ATF4 | CASP3 | 0.594 |
| ATM | CASP2 | 0.583 |
| APAF1 | NLRC4 | 0.58 |
| APAF1 | BMF | 0.576 |
| ATM | PMAIP1 | 0.567 |
| NLRC4 | NOD1 | 0.561 |
| CASP3 | DFNA5 | 0.556 |
| BMF | CASP3 | 0.551 |
| APAF1 | BCL10 | 0.548 |
| APAF1 | ATM | 0.52 |
| APAF1 | HSPB1 | 0.518 |
| APAF1 | NOD1 | 0.508 |
| DDIT3 | PMAIP1 | 0.505 |
| ATF4 | SAT1 | 0.489 |
| BCL10 | NLRC4 | 0.486 |
| CASP3 | KRT8 | 0.48 |
| ATF4 | SLC7A11 | 0.475 |
| BCL10 | NOD1 | 0.471 |
| ATM | E2F2 | 0.469 |
| RPL8 | TFRC | 0.469 |
| BAK1 | CASP2 | 0.462 |
| CASP2 | PMAIP1 | 0.46 |
| CASP3 | TFRC | 0.459 |
| BCL10 | PLCG1 | 0.456 |
| CASP2 | NLRC4 | 0.447 |
| TNFRSF12A | TNFRSF21 | 0.445 |
| HSPB1 | KRT8 | 0.438 |
| NOD1 | PMAIP1 | 0.433 |
| CASP3 | TNFRSF21 | 0.426 |
| BCAP31 | CASP3 | 0.414 |
| DFNA5 | NLRC4 | 0.414 |
| APAF1 | DDIT3 | 0.412 |
Protein-protein correlation scores of those genes that have p<0.05 correlation with the OS rate.

## Slide 21
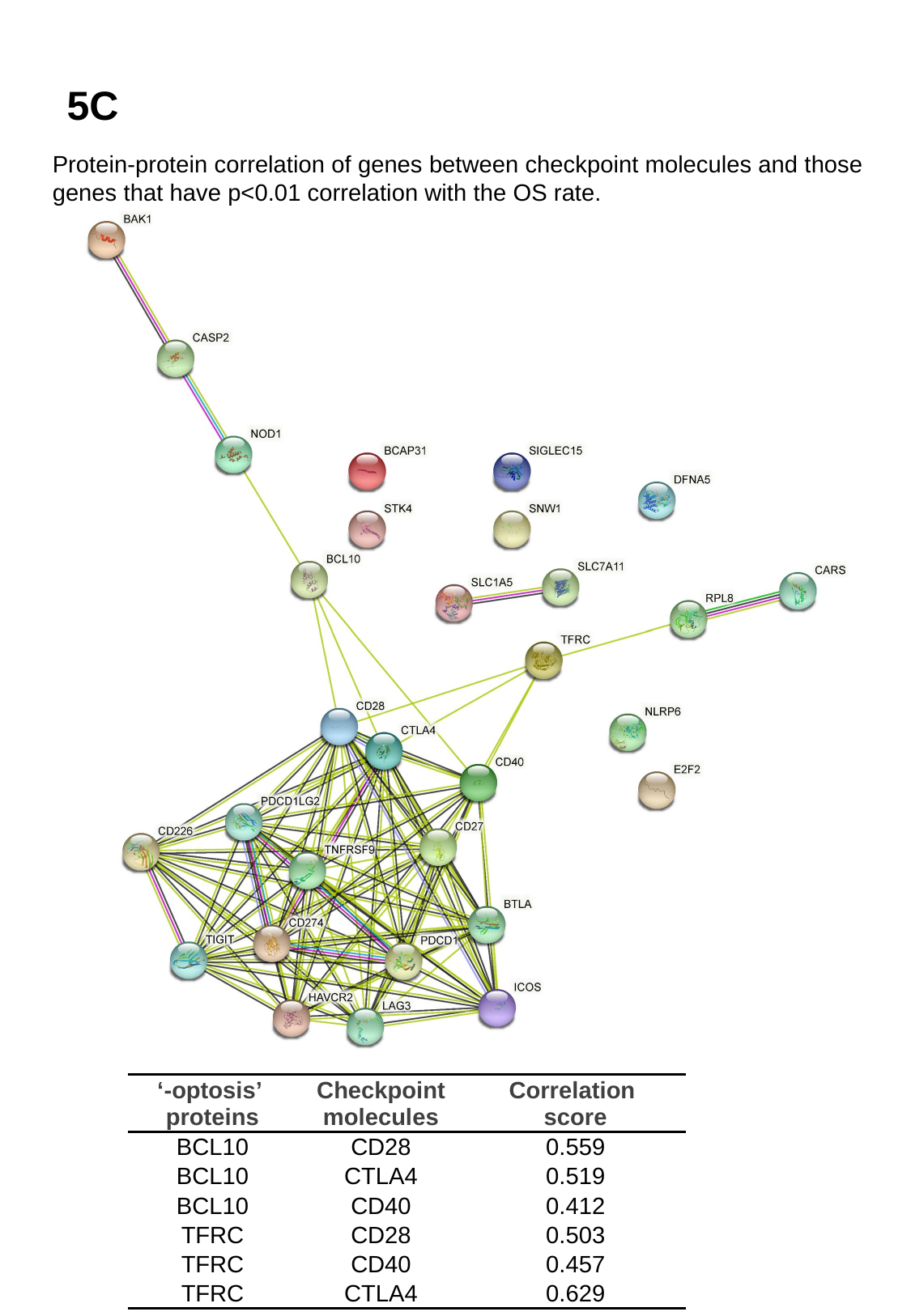

5C
Protein-protein correlation of genes between checkpoint molecules and those genes that have p<0.01 correlation with the OS rate.
| ‘-optosis’ proteins | Checkpoint molecules | Correlation score |
| --- | --- | --- |
| BCL10 | CD28 | 0.559 |
| BCL10 | CTLA4 | 0.519 |
| BCL10 | CD40 | 0.412 |
| TFRC | CD28 | 0.503 |
| TFRC | CD40 | 0.457 |
| TFRC | CTLA4 | 0.629 |

## Slide 22
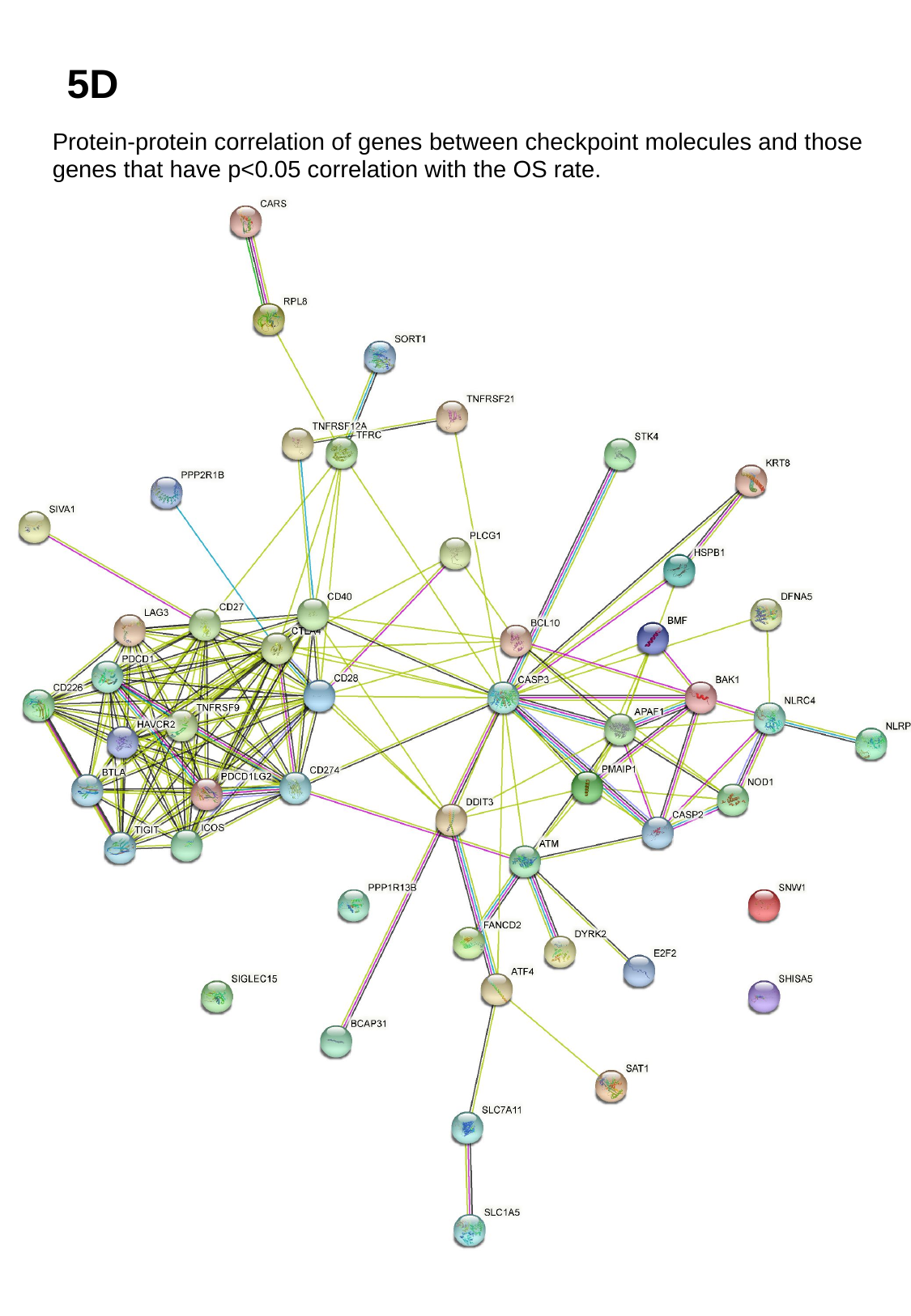

5D
Protein-protein correlation of genes between checkpoint molecules and those genes that have p<0.05 correlation with the OS rate.

## Slide 23
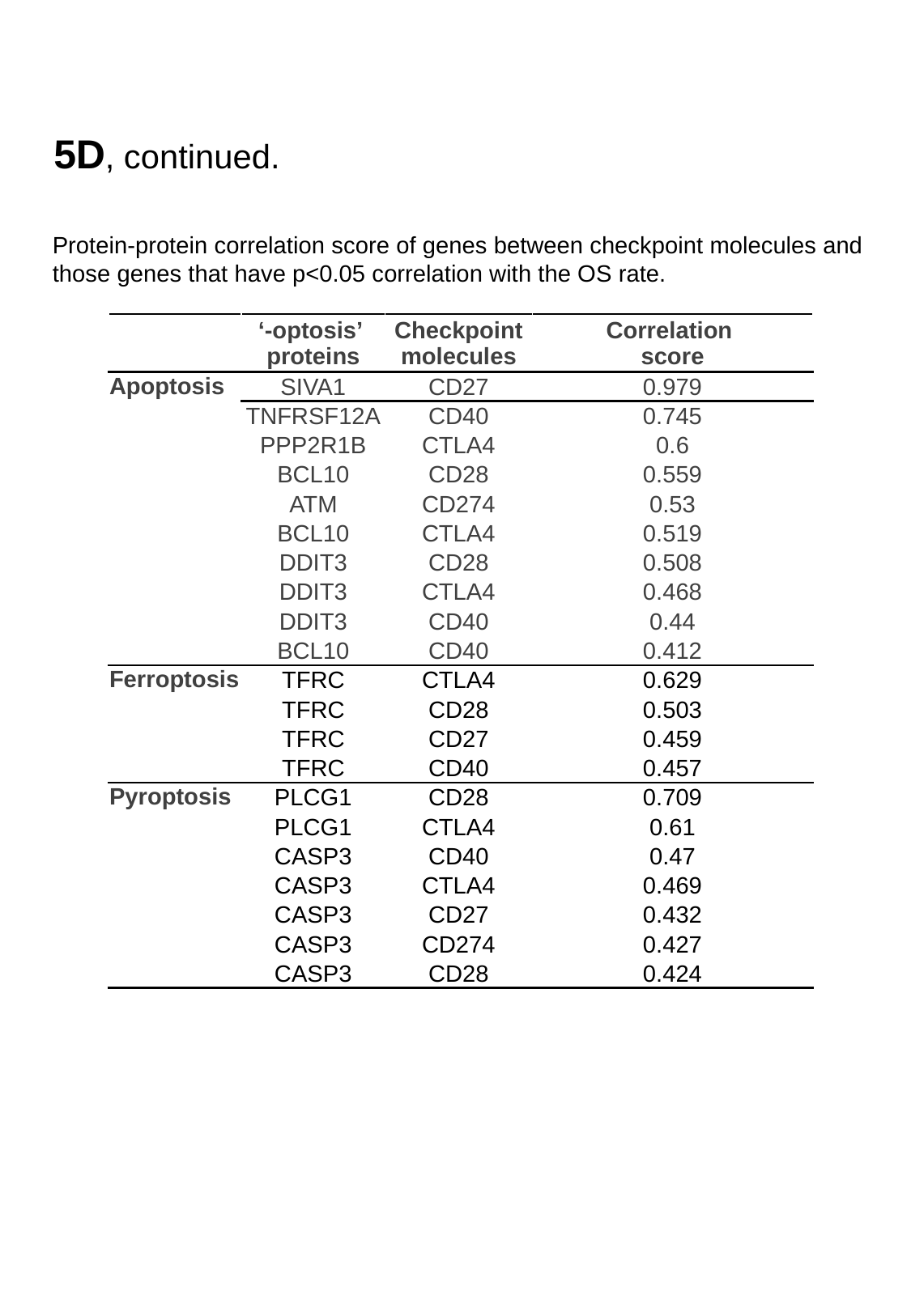

5D, continued.
Protein-protein correlation score of genes between checkpoint molecules and those genes that have p<0.05 correlation with the OS rate.
| | ‘-optosis’ proteins | Checkpoint molecules | Correlation score |
| --- | --- | --- | --- |
| Apoptosis | SIVA1 | CD27 | 0.979 |
| | TNFRSF12A | CD40 | 0.745 |
| | PPP2R1B | CTLA4 | 0.6 |
| | BCL10 | CD28 | 0.559 |
| | ATM | CD274 | 0.53 |
| | BCL10 | CTLA4 | 0.519 |
| | DDIT3 | CD28 | 0.508 |
| | DDIT3 | CTLA4 | 0.468 |
| | DDIT3 | CD40 | 0.44 |
| | BCL10 | CD40 | 0.412 |
| Ferroptosis | TFRC | CTLA4 | 0.629 |
| | TFRC | CD28 | 0.503 |
| | TFRC | CD27 | 0.459 |
| | TFRC | CD40 | 0.457 |
| Pyroptosis | PLCG1 | CD28 | 0.709 |
| | PLCG1 | CTLA4 | 0.61 |
| pyroptosis | CASP3 | CD40 | 0.47 |
| | CASP3 | CTLA4 | 0.469 |
| | CASP3 | CD27 | 0.432 |
| | CASP3 | CD274 | 0.427 |
| | CASP3 | CD28 | 0.424 |

## Slide 24
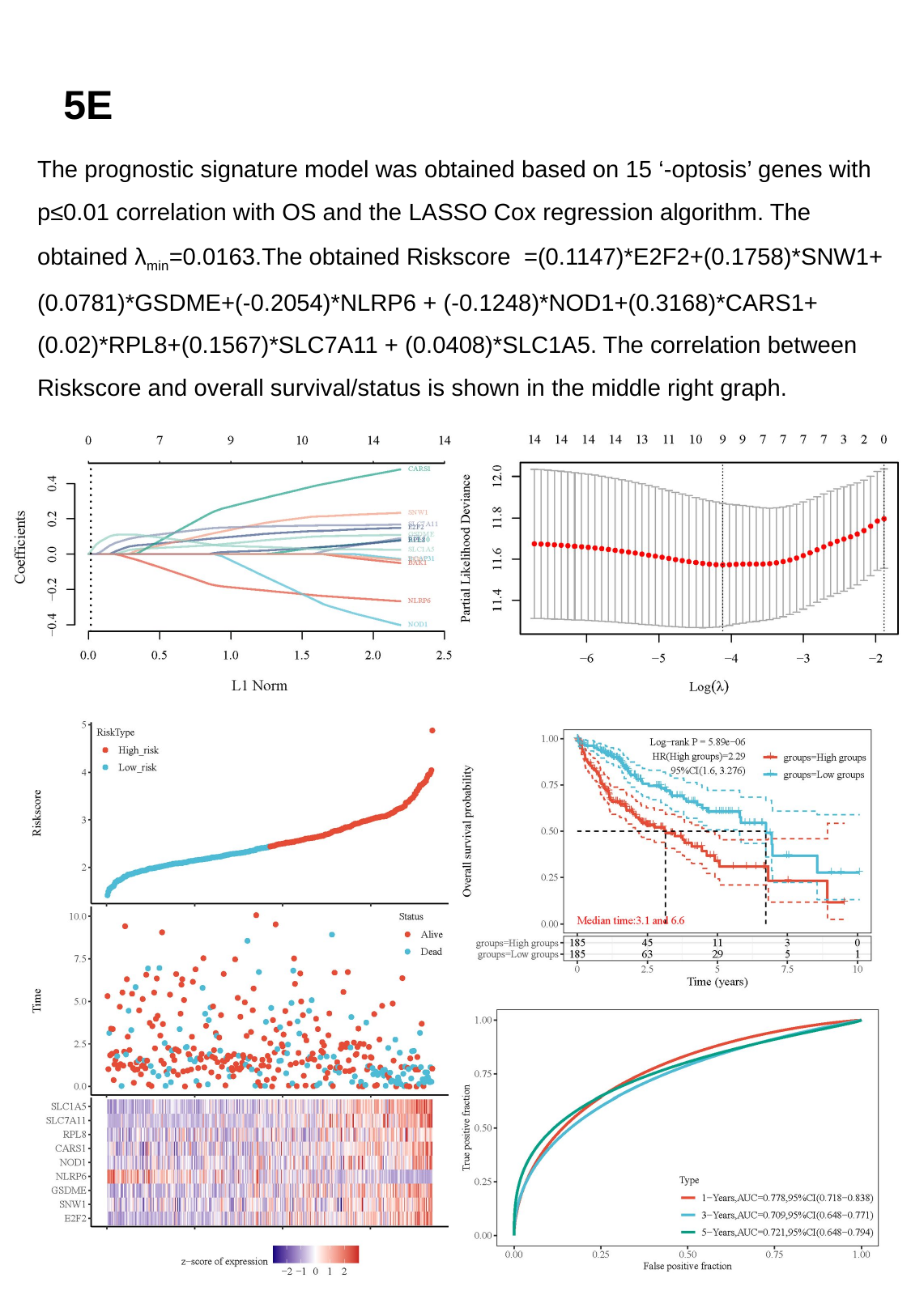

5E
The prognostic signature model was obtained based on 15 ‘-optosis’ genes with p≤0.01 correlation with OS and the LASSO Cox regression algorithm. The obtained λmin=0.0163.The obtained Riskscore =(0.1147)*E2F2+(0.1758)*SNW1+ (0.0781)*GSDME+(-0.2054)*NLRP6 + (-0.1248)*NOD1+(0.3168)*CARS1+ (0.02)*RPL8+(0.1567)*SLC7A11 + (0.0408)*SLC1A5. The correlation between Riskscore and overall survival/status is shown in the middle right graph.
